# Gut Microbiota-driven Tryptophan Metabolism Towards the Indole Pathway Mediates *Schisandra Chinensis* Polysaccharide's Alleviation of Ulcerative Colitis and Comorbid Depression via Aryl Hydrocarbon Receptor

**DOI:** 10.7150/ijbs.125012

**Published:** 2026-02-11

**Authors:** Jiuba Zhang, Shuai Yan, Ting Gao, Mingxuan Li, Yu Li, Lin Li, De Ji, Zhenhua Bian, Wei Huang, Jinjun Hou, Tulin Lu, Lianlin Su

**Affiliations:** 1 College of Pharmacy, Nanjing University of Chinese Medicine, Nanjing, 210023, China.; 2 Suzhou TCM Hospital Affiliated to Nanjing University of Chinese Medicine, Suzhou, 215009, China.; 3 Wuxi TCM Hospital Affiliated to Nanjing University of Chinese Medicine, Wuxi, 214071, China.; 4 Changzhou Hospital Affiliated to Nanjing University of Chinese Medicine, Changzhou, 213003, China.; 5 Shanghai Institute of Materia Medica, Shanghai, 200003, China.

**Keywords:** Ulcerative colitis and associated depression, *Schisandra Chinensis* polysaccharide, Tryptophan metabolic homeostasis, Indole-3-propionic acid, Aryl hydrocarbon receptor

## Abstract

Patients with ulcerative colitis (UC) exhibit heightened depression risk, linked to microbiota-gut-brain axis dysfunction. This study isolated a novel low-molecular-weight *Schisandra chinensis* polysaccharide (SCP) that ameliorated UC and comorbid depression by remodeling gut microbiota, redirecting tryptophan (Trp) metabolism toward the indole pathway, and activating aryl hydrocarbon receptor (AhR). Structurally, SCP features a →4)-α-D-Glc*p* backbone with *O*-6 branched chains. In dextran sulfate sodium-induced UC mice, SCP mitigated colonic inflammation, restored intestinal barrier integrity, and improved depression-like behaviors by repairing blood-brain barrier, reducing neuroinflammation, preserving hippocampal neurons, and modulating synaptic plasticity. Multi-omics revealed SCP enriched beneficial microbiota (e.g., *Limosilactobacillus reuteri*) and rebalanced Trp metabolism along the gut-brain axis. SCP suppressed the hyperactive kynurenine (Kyn) pathway (reduced Kyn/Trp ratio) while elevating indole-3-propionic acid (IPA) levels in colon, serum, and hippocampus. Functioning as a pivotal molecule, IPA exerted dual anti-inflammatory effects in both colon and hippocampus via AhR activation and NF-κB inhibition. Antibiotic depletion and fecal microbiota transplantation validated SCP's microbiota-dependent efficacy, while IPA supplementation recapitulated SCP's benefits. AhR inhibition abolished SCP's therapeutic actions, confirming AhR as the critical target. Collectively, these findings propose a novel therapeutic strategy for UC and associated depression, highlighting SCP's potential value in targeting the Trp metabolism-AhR axis.

## Introduction

Ulcerative colitis (UC), a chronic relapsing inflammatory bowel disease (IBD), is characterized by persistent colonic mucosal inflammation manifesting clinically as abdominal pain, diarrhea, and hematochezia 1. Emerging evidence suggests UC pathophysiology extends beyond intestinal lesions to encompass diverse extraintestinal manifestations and complications. Epidemiological studies reveal significantly elevated psychiatric comorbidities in UC patients, particularly depression, with prevalence rates reaching 15%-30% compared to 9.8%-15.8% in the general population 2. Animal model studies demonstrate that fecal microbiota transplantation (FMT) from UC patients or colitis mice to healthy recipients induced depression-like behaviors 3, while dextran sodium sulfate (DSS)-induced UC mice exhibit characteristic depressive phenotypes 4, 5. This gut-brain comorbidity underscores the imperative to elucidate underlying mechanisms and develop targeted therapeutics.

The microbiota-gut-brain axis serves as a pivotal bidirectional communication pathway linking the gastrointestinal and central nervous systems (CNS). Under physiological conditions, gut microbiota and their metabolites maintain intestinal homeostasis, whereas dysbiosis triggers pro-inflammatory cytokines release (e.g., TNF-α, IL-1β), compromising tight junction (TJ) and mucosal integrity, leading to "leaky gut" 6, 7. Subsequent translocation of inflammatory mediators and toxins through the impaired intestinal barrier initiates systemic inflammation and disrupts blood-brain barrier (BBB) integrity 8. Concurrent increases in intestinal permeability and CNS barrier dysfunction facilitate the infiltration of gut-derived molecules into the brain, promoting neuroinflammation. Persistent neuroinflammation disrupts neurons and synaptic plasticity, ultimately precipitating depressive symptoms 9. Tryptophan (Trp), a crucial molecular link in gut-brain crosstalk, exerts regulatory effects via circulatory, vagal, and immune-mediated pathways through its metabolites. Trp metabolism occurs through three competing pathways: the serotonin (5-HT) pathway, the kynurenine (Kyn) pathway, and the gut microbiota-mediated indole pathway 10. Dysregulated Trp metabolism is recognized as a hallmark of microbiota-gut-brain axis dysfunction. For instance, gut dysbiosis and markedly reduced levels of indole-3-propionic acid (IPA), a tryptophan-derived metabolite, in feces and blood were observed in 16p11.2^+/-^ autism model mice 11. Importantly, UC-associated inflammation robustly induces indoleamine 2,3-dioxygenase 1 (IDO1) expression, skewing Trp metabolism toward the kyn pathway. This metabolic shift promotes the accumulation of neurotoxic metabolites such as 3-hydroxykynurenine and quinolinic acid (QA), while suppressing protective indole pathway metabolites 12. Indole derivatives like IPA and indole-3-acetic acid demonstrate anti-inflammatory properties, enhance intestinal barrier function, and exert neuroprotective effects by activating the aryl hydrocarbon receptor (AhR) after crossing the BBB 13. AhR, a ligand-activated transcription factor, suppresses pro-inflammatory NF-κB signaling, maintains BBB homeostasis, and promotes neuronal survival. Given the central role of the microbiota-gut-brain axis in UC-associated depression, therapeutic strategies targeting this axis-particularly restoring Trp metabolic balance-hold substantial promise.

In recent years, natural polysaccharides have emerged as a research focus for disease intervention due to their potential to modulate the microbiota-gut-brain axis. *Schisandra chinensis* (SC), a traditional medicinal and edible resource documented in Chinese medical classics for its dual effects of "astringing intestines to relieve diarrhea" and "calming the mind to soothe anxiety," suggests potential in modulating gut-brain interactions 14. Modern pharmacological studies have identified polysaccharides as its primary active components, demonstrating anti-inflammatory, immunomodulatory, and neuroprotective properties 15. Our previous studies demonstrated that SC polysaccharide extracts significantly alleviated UC in C57BL/6 mice by restoring gut microbiota diversity and repairing intestinal barrier function 16. Additionally, emerging evidence indicated its capacity to mitigate neuropsychiatric disorders, particularly depression, by attenuating neuroinflammation 17. These independent findings highlight SC polysaccharides' dual protective roles in intestinal and neural systems, positioning them as an ideal therapeutic candidate for UC and its comorbid depression.

Building upon this foundation, we isolated a novel homogeneous SC polysaccharide (SCP) characterized by a →4)-α-D-Glcp backbone with O-6 branching. Using a DSS-induced chronic UC mouse model, we comprehensively evaluated its therapeutic efficacy against UC symptoms and comorbid depressive behaviors. Through integrative multi-omics analysis (metagenomics, spatial/untargeted/targeted metabolomics, transcriptomics) combined with antibiotic cocktail (ABX)-induced microbiota depletion, FMT, IPA supplementation, and AhR inhibition experiments, we demonstrated that SCP restored gut microbiota homeostasis and rebalanced Trp metabolism along the gut-brain axis. Specifically, SCP suppressed IDO1-mediated Kyn pathway hyperactivity while enhancing IPA production. Critically, by administering fluorescein isothiocyanate (FITC)-labeled IPA via intraperitoneal injection, we further confirmed its ability to cross the BBB. The accumulated IPA activated AhR signaling to inhibit NF-κB-driven inflammation in both colonic and hippocampal tissues, thereby concurrently ameliorating UC and depressive phenotypes. These findings provide novel mechanistic insights and therapeutic strategies for UC-related neuropsychiatric comorbidities.

## Materials and Methods

### Extraction, purification, and structural characterization of SCP

A total of 400 g of SC was reflux-extracted twice with 3200 mL water, each extraction lasting 3 hours. The extract was concentrated to 200 mL, mixed with four volumes of anhydrous ethanol, and stored at 4 ℃ for 24 h. Proteins were removed using the sevage method (polysaccharide solution to sevage reagent ratio = 5:1). The crude polysaccharide was fractionated via ion-exchange chromatography on a DEAE Sepharose FF column eluted with stepwise gradients of NaCl solutions (dH_2_O, 0.1 M, 0.2 M, and 0.5 M). Polysaccharide content was monitored using the anthrone-sulfuric acid method, and a polysaccharide elution profile was generated. The collected fractions were dialyzed (3.5 kDa cutoff) and lyophilized. Further purification was achieved through gel filtration column chromatography using a Chromdex 75PG column eluted with water at a flow rate of 1.5 mL/min. This process was repeated multiple times to enrich and purify the polysaccharides.

Qualitative analysis of SCP was performed using UV-Vis spectrophotometry. The polysaccharide solution was scanned across the 190-400 nm wavelength range. An absorption spectrum was plotted with wavelength as the abscissa and absorbance as the ordinate. SCP (2 mg) was mixed with KBr powder (200 mg) in a mortar, ground thoroughly, and pressed into a tablet for analysis using a fourier-transform infrared microspectrometer. The molecular weight of SCP was determined by high performance gel permeation chromatography (HPGPC). A 5 mg/mL polysaccharide solution was prepared in 0.05 M NaCl and analyzed using tandem columns (Ohpak SB-804 HQ and SB-806M HQ, 8 × 300 mm) with 0.05 M NaCl as the mobile phase at a flow rate of 0.50 mL/min. The column temperature was maintained at 40 ℃, and the injection volume was 30 μL. A calibration curve was constructed by plotting the logarithm of molecular weights of standard dextrans against their retention times. Monosaccharide composition was analyzed via high-performance liquid chromatography with an Agilent ZORBAX Eclipse XDB-C_18_ column. Standard monosaccharides-rhamnose, arabinose, galactose, glucose, xylose, mannose, galacturonic acid, glucuronic acid, glucosamine hydrochloride, and galactosamine hydrochloride (5 mg each)-and fucose (10 mg) were used to prepare a mixed standard stock solution. Samples were hydrolyzed with 2 M TFA (121 ℃, 2 h), derivatized, and eluted with acetonitrile: phosphate buffer (17:83, v/v) at 0.8 mL/min. Methylation was performed by dissolving 1 mg SCP in DMSO with 30 mg NaOH (30 min incubation), followed by iodomethane addition. The product was extracted with dichloromethane, hydrolyzed with 2 M TFA (121 ℃, 2 h), reduced with NaBD_4_, acetylated, and analyzed via GC-MS (7890A/5977B, Agilent) with temperature programming (50 ℃ to 230 ℃). For NMR, lyophilized SCP was dissolved in D_2_O, and ^1^H, ^13^C, COSY, HSQC, HMBC, and NOESY spectra were acquired on a 600 MHz Bruker spectrometer.

### Animal experiments

All animal procedures were authorized by the Institutional Animal Ethics Committee of Nanjing University of Chinese Medicine (Approval Code: 202311A048), adhering to the Laboratory Animal Management Guidelines in China. Male C57BL/6 mice aged six weeks were obtained from Jiangsu Jicui Yaokang Biotechnology Co., Ltd. [SCXK (SU)-2020-0004] (Nanjing, China), and were housed under standard conditions (25 ± 2 ℃, 12 h light/dark).

Experiment 1: After 7 days of acclimatization, mice were randomized into control (CON), DSS, mesalazine (5-ASA) (300 mg/kg), sulfasalazine (SASP) (200 mg/kg), low-dose SCP (SCPL) (200 mg/kg), and high-dose SCP (SCPH) (800 mg/kg) groups. Except for the CON group, UC modeling was induced in all other groups by administering 2% DSS solution for 5 days, followed by 5 days of distilled water. This constituted one cycle, which was repeated for four consecutive cycles. Starting on day 33, SCP, 5-ASA and SASP were administered daily via oral gavage to their respective groups for 2 weeks, while the CON and DSS groups received an equivalent volume of normal saline. Behavioral assessments were conducted over 7 days after treatment. Upon completion, all animals were euthanized, and colon (photographed immediately), brain, and hippocampus tissues were dissected. Sections of colon and brain were preserved for histopathological and immunofluorescence analyses; remaining samples were stored at -80 ℃ for further investigation. Fecal samples were collected for gut microbiota profiling, and serum was obtained for metabolomic and enzyme-linked immunosorbent assay (ELISA).

Experiment 2: After 7 days of acclimatization, mice were randomly divided into four groups: CON, DSS, ABX+SCP, and FMT. Except for the CON group, all mice underwent DSS-induced UC modeling using the protocol described in Experiment 1. Following model establishment, the ABX+SCP and FMT groups received daily oral administration (200 μL) of ABX for 10 days. The ABX consisted of vancomycin (100 mg/kg), neomycin (200 mg/kg), metronidazole (200 mg/kg), and ampicillin (200 mg/kg). Fecal samples were collected before and after ABX treatment to assess gut microbiota depletion. After ABX cessation, mice underwent a 3-day intestinal washout phase. Fecal samples (100 g) from SCP-treated mice were homogenized in 1 mL of sterile PBS, vortexed for 5 min, and centrifuged at 12000 rpm for 5 min to collect the supernatant. Subsequently, ABX+SCP and FMT groups received daily interventions of SCP (800 mg/kg) or FMT (200 μL), respectively, for two weeks. Post-treatment fecal samples from the FMT group were analyzed for microbiota reconstitution. Behavioral tests were conducted during the final 7 days, after which mice were euthanized. Colon, serum, hippocampal, and brain samples were harvested and stored as outlined in Experiment 1.

Experiment 3: After 7 days of acclimatization, mice were randomly assigned to three groups: CON, DSS, and IPA. The UC model was induced in all groups except the CON group using a four-cycle DSS protocol. Starting on day 33, the IPA group received daily oral administration of IPA (20 mg/kg) for 14 consecutive days. After the intervention period, behavioral assessments were conducted over 7 days. All animals were subsequently euthanized, and colon, serum, hippocampal, and brain samples were collected as previously described.

Experiment 4: After 7 days of acclimatization, mice were randomly divided into four groups: CON, DSS, SCP, and SCP+AhR. UC model was induced in all groups except the CON group using a four-cycle DSS protocol. Starting on day 33, the SCP group initiated daily oral treatment with SCP (800 mg/kg), while the SCP + AhR group received co-administration of SCP (800 mg/kg) and the AhR inhibitor (CH-223191, 10 mg/kg) for 14 days. After the intervention period, behavioral assessments were conducted over 7 days. All animals were subsequently euthanized, and colon, serum, hippocampal, and brain samples were collected as previously described.

Experiment 5: After 7 days of acclimatization, mice were randomly assigned to three groups: CON, DSS, and FITC-IPA. The UC model was induced in all groups except the CON group using a four-cycle DSS protocol. On the terminal day, the FITC-IPA group were administered a single intraperitoneal injection of IPA (20 mg/kg). After a 2-hour interval, all animals were euthanized, and brain tissue samples were collected as described previously.

### Disease activity index (DAI) and colonic mucosal damage index (CMDI) assessment

The DAI was obtained by averaging the total points from three parameters: body weight reduction, fecal characteristics, and hemorrhagic manifestations. For body weight evaluation, progressive percentage reductions were quantified as: 0 (< 1%), 1 (1~5%), 2 (5~10%), 3 (10~20%), and 4 (> 20%) points. Occult blood involved a pyramidon-based fecal occult blood kit testing, with 0 points for negative results, 2 points for occult blood detection, and 4 points for visible bleeding. Additionally, solid feces scored 0, loose stools received 2 points, and liquid feces were allocated 4 points. The CMDI was assessed based on mucosal appearance: normal tissue (0), mild hyperemia (1), hyperemia with minor ulceration (2), ulcerative lesions < 1 cm (3), and extensive ulceration exceeding 1 cm (4).

### Behavioural tests

Morris Water Maze (MWM): A circular platform was placed in the fourth quadrant, submerged 1 cm below the water surface. During the 4-day training period, the escape latency threshold for mice was set at 60 s. Mice reaching the platform within this time were recorded as escape latency, while those failing to locate the platform were guided to it and allowed to remain for 20 s. On day 5, the platform was removed, and mice were introduced into the water from the second quadrant. Behavioral parameters were recorded over 1 min. Open Field Test (OFT): Prior to testing, mice were acclimatized to the experimental environment for 30 minutes. Each mouse was then gently removed from its cage and consistently placed in the same corner of the testing arena. Locomotor activity within the open field was monitored for 5 minutes. Tail Suspension Test (TST): Mice were acclimatized to the experimental environment for 30 minutes before testing. Each mouse was gently restrained and rapidly gripped with a clamp approximately 1~2 cm from the tip of its tail. The mouse was immediately suspended from a horizontal bar, ensuring complete suspension. Video recording commenced, and immobility time was calculated starting from the second minute over a 6-minute observation period. Forced Swim Test (FST): Mice were acclimatized to the experimental environment for 30 minutes prior to testing. A cylindrical container was filled with water (22 ± 2 ℃), ensuring the hind limbs could not reach the bottom and the tail could not contact the sidewalls. A video recording device was positioned directly in front of the apparatus to capture mouse behavior during the 6-minute test. Immobility time was quantified starting from the second minute of the recording period.

### Hematoxylin and eosin (HE), alcian blue, and nissl staining

Colonic and brain tissues underwent post-fixation with 4% paraformaldehyde, subsequently embedded in paraffin, and sliced into 4-μm sections. HE staining was applied to examine histopathological alterations using a microscope. Colonic histopathological scores were assigned as follows: 0 points (no lamina propria elevation), 1 point (mild lamina propria elevation), 2 points (moderate lamina propria elevation with inflammatory cell infiltration), 3 points (marked lamina propria elevation with extensive perinuclear inflammatory infiltration), and 4 points (transmural inflammation). For quantitative analysis, alcian blue staining (to visualize goblet cells) and nissl staining (to assess neuronal density) were conducted using alcian blue solution and nissl stain, respectively.

### Immunofluorescence staining and microscopic observation

Colon and brain tissues were fixed in 4% paraformaldehyde, embedded in paraffin, and sectioned. For immunofluorescence staining, antigen retrieval was conducted in antigen retrieval buffer, followed by blocking with 3% hydrogen peroxide and 3% BSA. Colonic tissue sections were incubated with the first primary antibody (Occludin, 1:2000 dilution) at 4 ℃ overnight, followed by TSA-conjugated fluorescence labeling. Sequential staining was performed using the second (Claudin-5, 1:1000) and third (ZO-1, 1:1000) primary antibodies under identical conditions. Brain tissue sections were incubated with the primary antibody (NeuN, 1:2000), and both tissue types were also stained with an anti-AhR antibody (1:2000). For direct observation of FITC-IPA, brain tissue sections were processed without antigen retrieval, blocking, or antibody incubation steps. Nuclei were counterstained with DAPI (10 min, protected from light). Finally, slides were mounted with anti-fade medium and imaged using a fluorescence microscope.

### Measurement of inflammatory cytokine levels

Levels of TNF-α, IL-1β, IL-4, IL-6, and IL-10 in colon, brain, and serum samples were quantified using mouse-specific ELISA kits following the manufacturer's instructions.

### Transmission electron microscopy (TEM) analysis

The brain tissues were dissected into cubic segments (1 mm^3^) and promptly immersed in electron microscopy fixative. Secondary fixation was subsequently performed using 1% osmium tetroxide in 0.1 M PBS solution. Sequential dehydration was implemented through an ethanol series (50%, 70%, 80%, 90%, 95%, and 100%). Following resin embedding, ultrathin sections (60-80 nm) were prepared and dual-stained with uranyl acetate and lead citrate. Ultrastructural examination of BBB integrity and synaptic architecture was conducted using TEM.

### Metagenomic

Fresh fecal samples from mice were immediately preserved in sterile tubes following defecation. DNA extraction was performed using the FastPure Stool DNA Isolation Kit, and DNA integrity was assessed. Metagenomic sequencing was conducted on the Illumina NovaSeq^TM^ X Plus sequencing platform (Illumina, USA). All predicted gene sequences from samples were clustered with CD-HIT (http://weizhongli-lab.org/cd-hit/) to build a non-redundant gene catalog. Amino acid sequences from the non-redundant gene catalog were aligned against the NR database using Diamond (https://github.com/bbuchfink/diamond) to obtain taxonomic annotations and abundance data.

### Metabolomics

Untargeted metabolomics: Colon tissue (50 mg) was homogenized with 450 μL methanol (10 min), followed by centrifugation at 12000 rpm (4 ℃). Supernatants from both tissue lysates and serum samples (100 μL) were mixed with 400 μL of methanol, vortexed for 5 minutes, and centrifuged under identical conditions. The resulting supernatants were filtered using 0.22 μm membranes prior to analysis. Separation was achieved on a ZORBAX Extend-C_18_ column (2.1 mm × 100 mm, 1.8 μm) with a mobile phase comprising 0.1% aqueous formic acid (A) and acetonitrile (B). The gradient profile was set as: 0~2 min, 5~29% B; 2~10 min, 29~41% B; 10~12 min, 41~60% B; 12~20 min, 60~77% B; 20~25 min, 77~95% B. Ionization voltage was performed at 5500 V/-4500 V. Full-scan mass spectra were acquired in the range of m/z 100~1500 Da.

Targeted metabolomics: Standard solutions of Trp and related metabolites were prepared in 10% methanol. For serum samples, aliquots were successively mixed with 100 μL and 900 μL of 80% methanol, vortexed, and centrifuged (4 ℃, 12000 rpm, 5 min). The supernatant (100 μL) was spiked with 20 ppb Trp-d5 (1:1, v/v) before analysis. Tissue samples were homogenized in 100 μL of 80% methanol (55 Hz, 60 s), supplemented with 900 μL of 10% methanol, and homogenized again. After centrifugation under the same conditions, the supernatant was diluted fivefold with 10% methanol and similarly mixed with internal standard. Chromatographic separation was performed on an ACQUITY UPLC® HSS T3 column (2.1 × 150 mm, 1.8 μm) using 0.1% formic acid in water (A) and 0.1% formic acid in methanol (B) with the following gradient: 10% B (0~0.5 min), 10~30% B (0.5 ~ 2 min), 60% B (2 ~ 3 min), 60~98% B (3 ~ 6 min), 98% B (6 ~ 7.5 min), then re-equilibrated to 10% B until 9 min.

Spatial metabolomics: Mass spectrometry imaging data acquisition was conducted using a line-by-line scanning mode, with X- and Y-axis scanning lengths determined according to section dimensions for systematic regional sampling. After completion of each line scan, the sample stage rapidly returned to the scanning origin at 10 mm/s to prevent potential contamination or damage to unscanned areas. The platform subsequently advanced incrementally along the Y-axis by a predetermined step distance prior to initiating the next scanning cycle, repeating this protocol until full tissue coverage was achieved. For both positive and negative ion detection modes, the spray solvent composition was maintained at 80:20 (v/v) acetonitrile: water delivered at 2.0 μL/min. Critical instrumental parameters included: nebulizing gas pressure 0.6 MPa, spray angle 60°, nozzle-to-section distance 3 mm, nozzle-to-transfer tube distance 5 mm, transfer tube-to-orifice distance 2 mm, and ion source temperature stabilized at 150 ℃.

### RNA-seq analysis

Total RNA was extracted from the tissue samples, with RNA concentration and purity measured using a Nanodrop2000 spectrophotometer, while RNA integrity was verified through agarose gel electrophoresis. High-throughput sequencing was performed on the NovaSeq X Plus platform, and gene expression levels were quantified as transcripts per million (TPM). Bioinformatic analyses including volcano plots, heatmaps, Gene Ontology (GO) enrichment, and Kyoto Encyclopedia of Genes and Genomes (KEGG) pathway analyses were conducted using the Majorbio Cloud Platform tools (https://www.majorbio.com/tools).

### Quantitative real-time PCR

Total RNA was extracted from colon and hippocampal tissues using TRIzol reagent according to the manufacturer's protocol. RNA quantity and purity were determined by measuring absorbance at 260 nm and 280 nm. RT-PCR was performed using a real-time fluorescence quantitative PCR instrument (LT6276, Gene Company) with SYBR Green master mix in a 20 μL reaction volume. Primer sequences are provided in Table S1. Relative gene expression levels were calculated using the -ΔΔCt method and normalized to GAPDH expression.

### Western blot

According to the manufacturer's instructions, total proteins were extracted from colon and hippocampal tissues using RIPA buffer containing 1% protease inhibitor cocktail. Protein concentration was quantified using the BCA protein assay kit. Protein separation was performed through SDS-PAGE, followed by electrophoretic transfer onto PVDF membranes. The membranes were incubated overnight at 4 °C with the following primary antibodies (rabbit polyclonal antibodies) at specified dilutions: Occludin (1:2500), Claudin-5 (1:5000), Muc2 (1:2000), AhR (1:5000), NF-κB p65 (1:5000), NF-κB p-p65 (1:5000), IDO1 (1:5000) postsynaptic scaffolding protein 95 (PSD-95) (1:1000), synaptophysin (SYN) (1:1000), GAPDH (1:10000), and α-tubulin (1:10000). Subsequently, the membranes were incubated with secondary antibodies (1:5000) for 2 h at room temperature. Protein bands were visualized using enhanced chemiluminescence reagent, and densitometric analysis was performed using ImageJ software.

### Cell culture and drug treatment

HT22 cells were cultured in DMEM/F-12 medium supplemented with 10% fetal bovine serum and 1% penicillin-streptomycin. The cells were seeded into 96-well plates and incubated for 12 hours, followed by grouping into three experimental conditions: CON, TNF-α group (20 ng/mL), and TNF-α+IPA group (5 μmol/L IPA + 20 ng/mL TNF-α). After 48 hours of incubation, cell viability and apoptosis levels were assessed using the CCK-8 assay and flow cytometry, respectively. Additionally, inflammatory cytokine levels were measured via ELISA.

### Statistical analysis

All datasets were analyzed for statistical significance using GraphPad Prism 8 (San Diego, USA), with results expressed as mean ± sem. Significant differences between two groups were evaluated using a two-tailed unpaired Student's t-test, whereas differences among multiple groups were analyzed using one-way or two-way analysis of variance (ANOVA) followed by Dunnett's post hoc multiple comparisons test. A *p*-value < 0.05 was considered statistically significant.

## Results

### Structural Characterization of SCP

**Figure 1:**
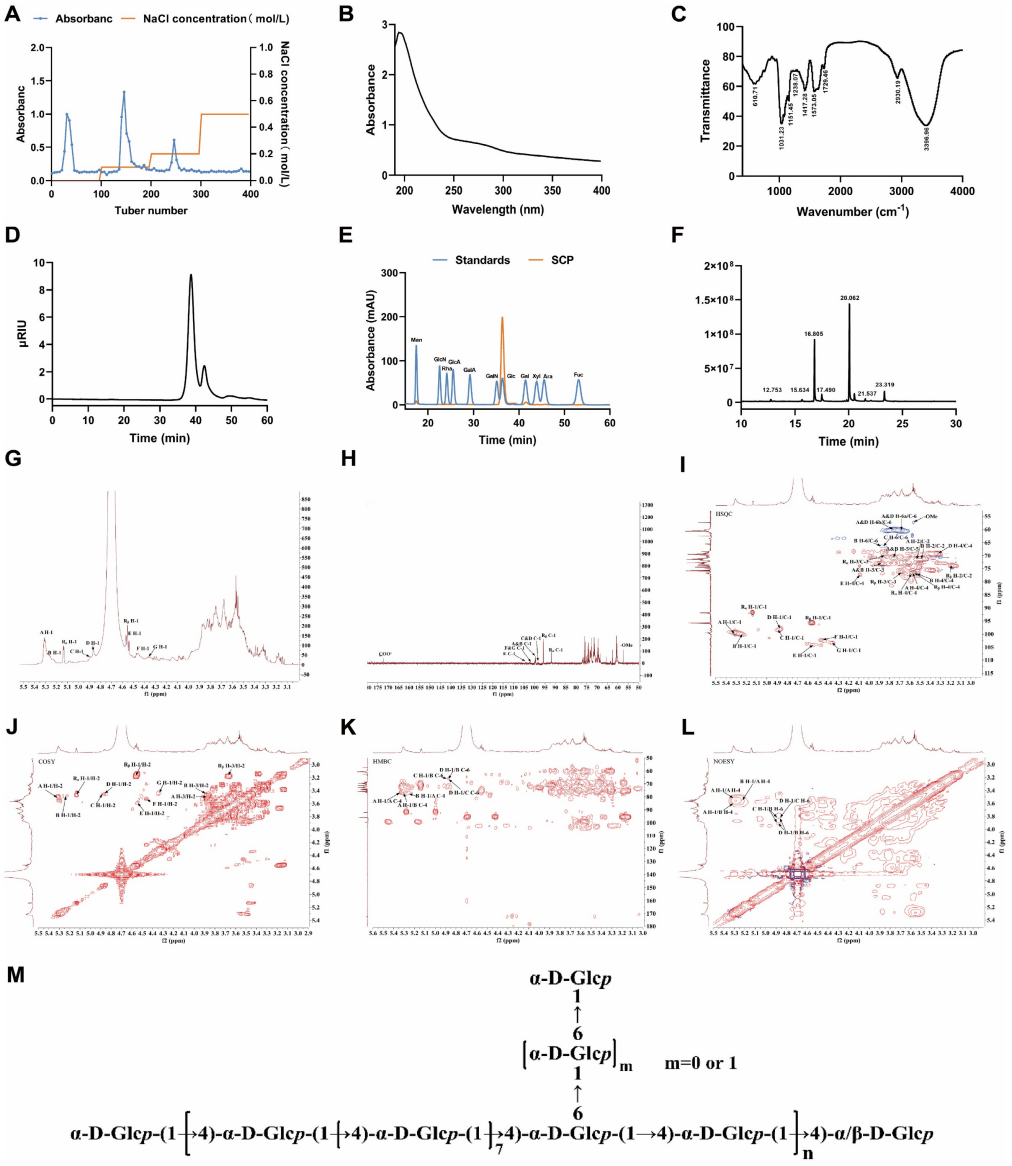
Structural characterization of SCP. **(A)** DEAE Sepharose FF elution profile, **(B)** UV-Vis spectroscopic analysis of SCP, **(C)** Infrared spectroscopic analysis of SCP, **(D)** HPGPC of SCP, **(E)** Monosaccharide composition analysis of SCP, **(F)** Methylation analysis of SCP, **(G)** ^1^H NMR spectrum, **(H)** ^13^C NMR spectrum, **(I)** HSQC spectrum, **(J)** COSY spectrum, **(K)** HMBC spectrum, **(L)** NOESY spectrum, **(M)** Proposed structure of SCP.

Three major fractions were obtained from ion-exchange column chromatography, as demonstrated by the elution profile in Fig. 1A. The 0.1 M NaCl-eluted fraction exhibited significantly higher polysaccharide content and yield compared to other fractions, which was subsequently purified through Chromdex 75PG column chromatography to isolate SCP. Ultraviolet absorption spectroscopy (Fig. 1B) revealed no characteristic peaks at 260 nm or 280 nm, confirming the absence of nucleic acids and protein contaminants. Fourier-transform infrared spectroscopy (Fig. 1C) displayed characteristic polysaccharide bands: a broad O-H stretching vibration at 3396.96 cm⁻¹, weak C-H stretching of methyl/methylene groups at 2930.19 cm⁻¹, and C-H bending vibrations between 1400-1200 cm⁻¹, thereby confirming SCP as a polysaccharide. HPGPC analysis revealed a single symmetric peak for SCP, confirming its homogeneity (Fig. 1D). The Mw of SCP was calculated as 2.26 kDa based on Pullulan standards. Monosaccharide composition analysis indicated that SCP predominantly consists of glucose (Fig. 1E). Methylation analysis was performed to elucidate glycosidic linkage patterns. Fig. 1F displayed the total ion chromatogram of SCP in methylation analysis, and Table S2 summarized the methylation data. SCP primarily contained two partially methylated alditol acetates: 1,4-Glcp and t-Glcp, consistent with monosaccharide composition results.

To further determine structural features, 1D NMR (^1^H, ^13^C) and 2D NMR spectra (COSY, HSQC, HMBC, NOESY) were acquired. The ^1^H NMR spectrum showed multiple anomeric proton signals at δ 4.3~5.5 ppm (Fig. 1G), while the ^13^C NMR spectrum exhibited anomeric carbon signals between δ 90~105 ppm, indicating the presence of several distinct sugar residues (Fig. 1H). Cross-peaks at δ 5.16/91.98 ppm (HSQC) and δ 5.13/3.46 ppm (COSY) for residue R_α_, as well as δ 4.55/95.69 ppm (HSQC) and δ 4.55/3.16 ppm (COSY) for residue R_β_ (Fig. 1I, J), indicated H-2/C-2 chemical shifts of δ 3.46/71.32 ppm and δ 3.16/73.86 ppm, respectively. By comparison with literature data, these reducing termini were identified as →4)-α-D-Glcp (R_α_) and →4)-β-D-Glcp (R_β_). The remaining proton and carbon signals were assigned using COSY/HSQC correlations and Table S3. The identified residues included: Residue A, →4)-α-D-Glcp-(1→; Residue B, →4,6)-α-D-Glcp-(1→; Residue C, →6)-α-D-Glcp-(1→; Residue D, α-D-Glcp-(1→; Residue E, →4)-β-D-Galp-(1→; Residue F, β-D-Galp-(1→; Residue G, →6)-β-D-Galp-(1→.

HMBC and NOESY spectra (Fig. 1K, L) revealed critical inter-residue correlations. A H-1/A C-4 (δ 5.30/76.75 ppm) and A H-1/A H-4 (δ 5.30/3.56 ppm) indicated →4)-α-D-Glcp-(1→4)-α-D-Glcp-(1→ linkages. A H-1/B C-4 (δ 5.30/76.74 ppm) and A H-1/B H-4 (δ 5.30/3.53 ppm) confirmed →4)-α-D-Glcp-(1→ linkages to →4,6)-α-D-Glcp-(1→ at O-4. B H-1/A C-4 (δ 5.26/76.75 ppm) and B H-1/A H-4 (δ 5.26/3.56 ppm) demonstrated reciprocal →4,6)-α-D-Glcp-(1→4)-α-D-Glcp-(1→ connections at O-4. D H-1/B C-6 (δ 4.86/66.51 ppm) and D H-1/B H-6 (δ 4.86/3.83, 3.87 ppm) established α-D-Glcp-(1→6)-α-D-Glcp-(1→ branching at O-6 of residue B. D H-1/C C-6 (δ 4.86/66.12 ppm) and D H-1/C H-6 (δ 4.86/3.85 ppm) confirmed α-D-Glcp-(1→6)-α-D-Glcp-(1→ linkages. C H-1/B C-6 (δ 4.88/66.51 ppm) and C H-1/B H-6 (δ 4.88/3.83, 3.87 ppm) suggested →6)-α-D-Glcp-(1→4,6)-α-D-Glcp-(1→ connections at O-6. Methylation analysis revealed a molar ratio of 9:1 for 1,4-Glcp and 1,4,6-Glcp residues, suggesting a backbone predominantly composed of →4)-α-D-Glcp-(1→ with O-6 branches. The 1,4,6-Glcp:1,6-Glcp ratio approximated 3:2. Due to low abundance, galactose residue linkages could not be conclusively determined. Based on these findings, the proposed structure of SCP is summarized in Fig. 1M.

### SCP alleviated DSS-induced UC by mitigating intestinal inflammation and restoring barrier integrity

**Figure 2:**
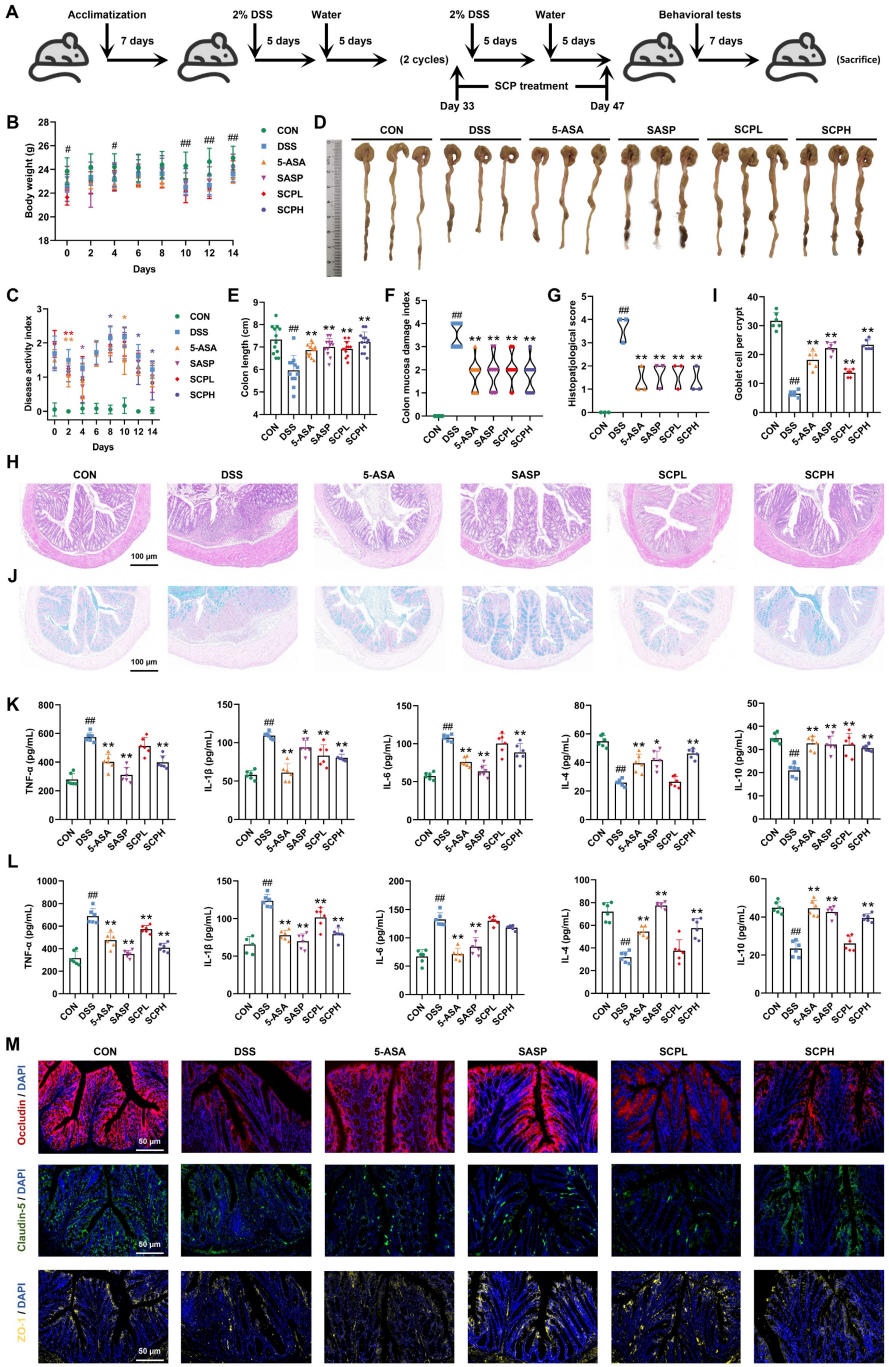
SCP treatment ameliorated DSS-induced UC in mice. A Experimental protocol timeline, B Body weight changes during SCP treatment (n=12/group), C DAI score changes during SCP treatment (n=12/group), D Representative images of colons from each group, E Statistical analysis of colon length (n=12/group), F Comparative CMDI scores among groups (n=12/group), G Histopathological scores of colon tissues based on HE staining (n=3/group), H Representative HE-stained colon sections, I Statistical analysis of goblet cell numbers (n=6/group), J Representative Alcian blue-stained colon sections, K ELISA detection of colonic inflammatory cytokine levels (n=6/group), L ELISA measurement of serum inflammatory cytokines (n=6/group), M Immunofluorescence analysis of tight junction protein expression in colonic tissues. ^#^*p* < 0.05, ^##^*p* < 0.01 versus CON group; ^*^p < 0.05, ^**^p < 0.01 versus DSS group.

As illustrated in Fig. 2A, a chronic UC mouse model was established with corresponding SCP treatment protocols. Compared to the CON group, DSS-induced UC mice exhibited significant weight loss at days 0, 4, 10, 12, and 14 (*p* < 0.05), which was effectively mitigated by SCP intervention (Fig. 2B). Throughout the experiment, the DAI scores in the DSS group were significantly higher than those in the CON group, confirming successful UC model establishment. After SCP treatment, UC mice exhibited significantly reduced DAI scores on days 2, 8, 12, and 14 (*p* < 0.05) (Fig. 2C). Notably, SCP demonstrated a stronger tendency than the positive drugs 5-ASA and SASP in reducing DAI scores. Colon length measurements (Fig. 2D, E) and CMDI scores (Fig. 2F) revealed that oral SCP effectively alleviated DSS-induced colon shortening and mucosal injury (*p* < 0.01). Histopathological evaluation via HE staining (Fig. 2G, H) further revealed that SCP dose-dependently attenuated inflammatory cell infiltration and crypt loss in DSS-treated mice, as reflected by reduced histopathological scores (*p* < 0.01). Goblet cells, critical components of the colonic epithelium responsible for mucin synthesis and secretion, play a pivotal role in maintaining intestinal barrier integrity 18. Alcian blue staining demonstrated that DSS exposure markedly reduced goblet cell numbers (*p* < 0.01) and disrupted their morphology in crypts, whereas SCP treatment effectively reversed these alterations (Fig. 2I, J). Additionally, SCP treatment normalized DSS-induced dysregulation of inflammatory cytokines in both colon and serum, including suppression of TNF-α, IL-1β, and IL-6 elevation, as well as restoration of IL-4 and IL-10 levels (Fig. 2K, L). This suggests that SCP may prevent systemic dissemination of inflammatory mediators by repairing intestinal barrier integrity. To validate this hypothesis, immunofluorescence staining was performed to assess TJ proteins (Occludin, Claudin-5, and ZO-1) in the intestinal barrier. Fluorescence intensity and area analysis revealed that SCP intervention markedly enhanced TJ protein expression in UC mice (Fig. 2M). Western blot analysis further corroborated that SCP significantly upregulated both TJ proteins and mucin (Muc2) expression (*p* < 0.05) (Fig. S1A).

### SCP ameliorated UC-associated depression by preserving BBB integrity, protecting hippocampal neurons, and modulating synaptic plasticity

**Figure 3:**
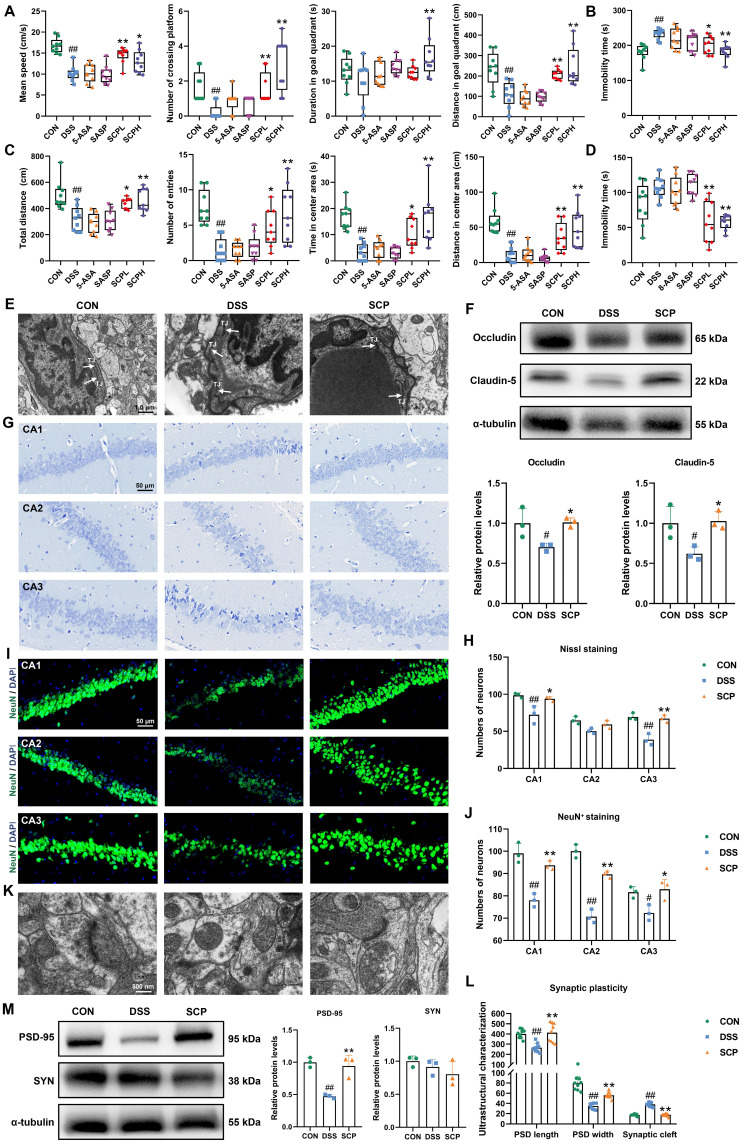
SCP treatment alleviated UC-associated depression. A Analysis of swimming speed, platform crossings, time spent in the target quadrant, and swimming distance in the target quadrant during the MWM (n=9/group), B Immobility time in the TST (n=9/group), C Total travel distance, entries into the central area, time spent in the central area, and distance traveled in the central area during the OFT (n=9/group), D Immobility time in the FST (n=9/group), E TEM images of BBB ultrastructure, F Western blot analysis of TJ proteins in the BBB (n=3/group), G Nissl staining to evaluate hippocampal neuronal damage, H Quantification of neuronal counts based on Nissl staining (n=3/group), I NeuN^+^ immunofluorescence staining for hippocampal neuronal activity, J Quantification of NeuN^+^ neuronal counts (n=3/group), K TEM images of synaptic ultrastructure, L Analysis of postsynaptic density length, width, and synaptic cleft (n=9/group), M Western blot analysis of synaptic plasticity-related protein expression (n=3/group). ^#^*p* < 0.05, ^##^*p* < 0.01 versus CON group; ^*^p < 0.05, ^**^p < 0.01 versus DSS group.

To evaluate the therapeutic potential of SCP in UC-associated depression, we conducted a series of behavioral tests, including the MWM, TST, OFT, and FST. In the MWM, mice in the DSS group demonstrated impaired spatial memory retention for platform localization compared to both the CON and SCP-treated groups (Fig. S1B). Specifically, SCP administration significantly improved swimming speed, platform crossings, duration in the target quadrant, and movement distance in the target quadrant (*p* < 0.05) (Fig. 3A). In the TST, SCP treatment markedly reduced immobility time in UC mice (*p* < 0.05) (Fig. 3B, and Fig. S1C). Trajectory analysis in the OFT revealed that SCP intervention augmented exploratory behavior in the central zone of UC mice (Fig. S1D), significantly increasing total travel distance, entries into the central zone, time spent in the central zone, and movement distance within the central zone (*p* < 0.05) (Fig. 3C). Similarly, in the FST, SCP administration significantly shortened immobility time in UC mice (*p* < 0.01) (Fig. 3D; Fig. S1E). Collectively, these findings demonstrated that SCP treatment effectively mitigated depression-like behaviors in UC mice.

Pro-inflammatory cytokines may translocate into systemic circulation through a compromised intestinal barrier, potentially disrupting the BBB and infiltrating the CNS 19. Given the critical role of BBB integrity in depression pathogenesis, TEM was employed to assess structural changes. UC mice exhibited diffuse disruption of TJ structures in the BBB, indicating impaired integrity. Strikingly, SCP treatment restored TJ architecture, yielding distinct and compact junctional bands (Fig. 3E). Western blot analysis further confirmed that oral SCP significantly reversed the downregulation of TJ proteins (Occludin and Claudin-5) expression (*p* < 0.05) (Fig. 3F). Concurrently, SCP intervention attenuated neuroinflammation in UC mice, as evidenced by decreased pro-inflammatory cytokines and elevated anti-inflammatory factors (*p* < 0.05) (Fig. S1F). CNS invasion of inflammatory cytokines exacerbates neuroinflammation, leading to hippocampal neuronal and synaptic injury 20, 21. As a key brain region involved in emotion and cognition, hippocampal pathology is strongly implicated in depression 22. Nissl staining (Fig. 3G, H) and NeuN^+^ immunofluorescence staining (Fig. 3I, J) revealed diminished neuronal activity and decreased hippocampal neuron counts in UC mice (*p* < 0.05), whereas SCP supplementation markedly improved these pathological changes, particularly in the CA1 and CA3 subregions. TEM-based ultrastructural analysis showed that UC mice exhibited significant reductions in postsynaptic density length and width, alongside widened synaptic clefts (*p* < 0.01), all of which were effectively reversed by SCP treatment (Fig. 3K, L). Furthermore, Western blot indicated that SCP restored synaptic plasticity by upregulating the expression of the excitatory PSD-95 (*p* < 0.05), while SYN expression remained unaltered (Fig. 3M).

### SCP restored the gut microbiota and reversed microbiota-mediated Trp metabolic imbalance in UC mice

**Figure 4:**
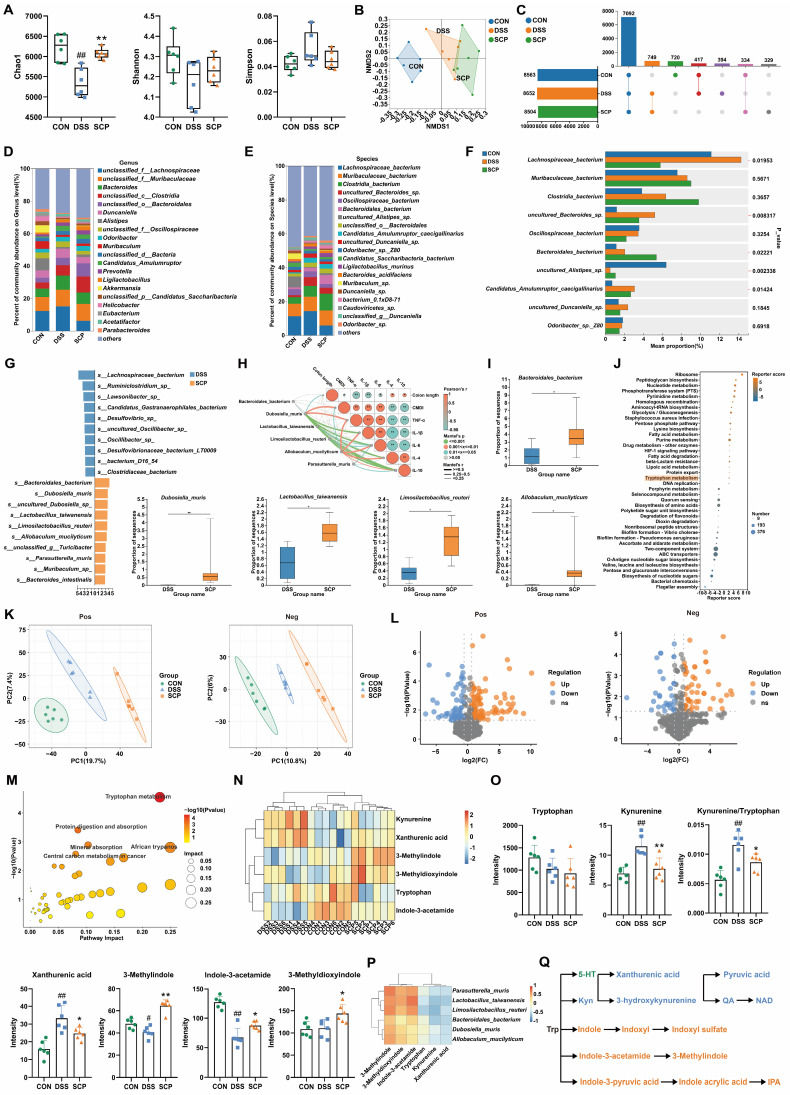
SCP reshaped gut microbiota and rebalanced Trp metabolism in UC mice. A Species-level α-diversity analysis (n=6/group), B Species-level β-diversity analysis, C Number of detected gut microbial species across groups, D Genus-level relative abundance analysis of gut microbiota, E Species-level relative abundance analysis of gut microbiota, F Top 10 differentially abundant microbial species, G LEfSe analysis (LDA > 2.5) identifying dominant microbial species (Top 20), H Mantel test analysis of correlations between gut microbiota and metabolites, I Significantly enriched gut microbial species in the SCP group, J KEGG pathway analysis highlighting functional alterations in gut microbiota, K OPLS-DA of colonic metabolomics, L Volcano plot showing differentially expressed metabolites, M KEGG enrichment analysis of colonic Trp metabolism pathways, N Hierarchical clustering heatmap of Trp derivatives across groups, O Comparative levels of Trp and its metabolites (n=6/group), P Correlation heatmap linking gut microbiota to Trp metabolism, Q Schematic of competitive Trp metabolic pathways. ^#^*p* < 0.05, ^##^*p* < 0.01 versus CON group; ^*^p < 0.05, ^**^p < 0.01 versus DSS group.

Having confirmed the ameliorative effects of SCP on UC and comorbid depressive behaviors, investigating its underlying mechanisms becomes essential. Given the central role of gut microbiota and their metabolites in the microbiota-gut-brain axis, integrated metagenomic and untargeted metabolomic analyses were conducted to evaluate global alterations. α-Diversity analysis revealed that DSS-treated mice exhibited reduced Chao1 and Shannon indices but increased Simpson index, indicating compromised gut microbial diversity (Fig. 4A). SCP intervention reversed this trend, with the Chao1 index showing particularly significant improvement (*p* < 0.05). Nonmetric multidimensional scaling analysis demonstrated that SCP administration markedly restored the overall structure of gut microbiota (Fig. 4B). Genus-level analysis aligned with these findings (Fig. S2A, B). Quantitatively, SCP treatment restored 72 genera (Fig. S2C) and 334 species (Fig. 4C). Phylum-level profiling indicated an elevated *Bacillota*/*Bacteroidota* ratio in UC mice, which was normalized by SCP intervention (Fig. S2D). At the genus level, SCP significantly modulated the abundance of *Bacteroides*, *Duncaniella*, *Alistipes*, *Candidatus_Amulumruptor*, *Muribaculum*, *Akkermansia*, *Parabacteroides*, and *Limosilactobacillus* (Fig. 4D; Fig. S2E). Species-level analysis further confirmed the restoration of *Lachnospiraceae_bacterium*, *Candidatus_Amulumruptor_caecigallinarius*, *Muribaculum_sp.*, *Candidatus_Saccharibacteria_bacterium*, and *Duncaniella_sp.* (Fig. 4E, F). Notably, Linear discriminant analysis (LDA) effect size (LEfSe) analysis identified eight SCP-enriched species (Fig. 4G), and Mantel tests revealed significant correlations between these species and UC-associated biomarkers (Fig. 4H). Among them, *Bacteroidales_bacterium*, *Dubosiella_muris*, *Lactobacillus_taiwanensis*, *Limosilactobacillus_reuteri*, and *Allobaculum_mucilyticum* exhibited pronounced increases in abundance in SCP-treated mice (*p* < 0.05) (Fig. 4I). Functional enrichment analysis using the KEGG database highlighted that amino acid metabolism, energy metabolism, and carbohydrate metabolism formed core interactive modules (Fig. S2F). Specifically, nucleotide metabolism, glycolysis/gluconeogenesis, lysine biosynthesis, and Trp metabolism were strongly associated with SCP's therapeutic effects (Fig. 4J).

Although metagenomic findings suggested alterations in Trp metabolism pathways, integrated metabolomic profiling was necessary to delineate colonic and systemic metabolic remodeling. Orthogonal partial least squares discriminant analysis (OPLS-DA) revealed that SCP treatment significantly ameliorated DSS-induced colonic metabolic dysregulation (Fig. 4K). Differential metabolite analysis (Fig. 4L) demonstrated that SCP intervention resulted in 156 upregulated and 49 downregulated metabolites (|FC| > 1.5, *p* < 0.05). KEGG enrichment analysis further identified Trp metabolism as a key pathway modulated by SCP, consistent with metagenomic findings (Fig. 4M). Among these differentially regulated metabolites, six Trp-related metabolites were identified, including Trp, Kyn, xanthurenic acid, indole-3-acetamide, 3-methylindole, and 3-methyldioxyindole. Cluster heatmaps visually illustrated their distinct expression patterns across CON, DSS, and SCP groups (Fig. 4N). SCP administration significantly reduced colonic Kyn and xanthurenic acid levels and the Kyn/Trp ratio, while elevating indole derivatives (3-methylindole, indole-3-acetamide, and 3-methyldioxyindole) in UC mice (*p* < 0.05) (Fig. 4O). Microbiota-metabolite correlation analysis further substantiated that these changes were closely associated with SCP-enriched bacterial species (Fig. 4P). The three primary pathways of Trp metabolism exhibit a dynamic competing relationship (Fig. 4Q). The reduced Kyn/Trp ratio indicated SCP-mediated suppression of the kyn pathway, whose hyperactivation has been shown to exacerbate neuroinflammatory responses. Concurrently, elevated indole derivatives indicated a metabolic shift toward the indole pathway. Furthermore, OPLS-DA revealed that SCP similarly reversed serum metabolic imbalances (Fig. S2G). Volcano plot analysis (Fig. S2H) identified 55 upregulated and 73 downregulated serum metabolites (|FC| > 1.5, *p* < 0.05), including four Trp derivatives: 3-hydroxykynurenamine, 3-methylindole, IPA, and indole-3-carboxylic acid. SCP treatment markedly downregulated the kyn pathway metabolite 3-hydroxykynurenamine while upregulating indole pathway metabolites 3-Methylindole, IPA, and Indole-3-carboxylic acid (*p* < 0.05) (Fig. S2I). KEGG analysis further confirmed substantial alterations in serum Trp metabolism (Fig. S2J). Collectively, SCP intervention effectively restored Trp metabolism homeostasis in both colon and serum, characterized by suppression of the kyn pathway and activation of the indole pathway.

### SCP mediated targeted suppression of hippocampal kyn pathway hyperactivation to promote IPA generation

**Figure 5:**
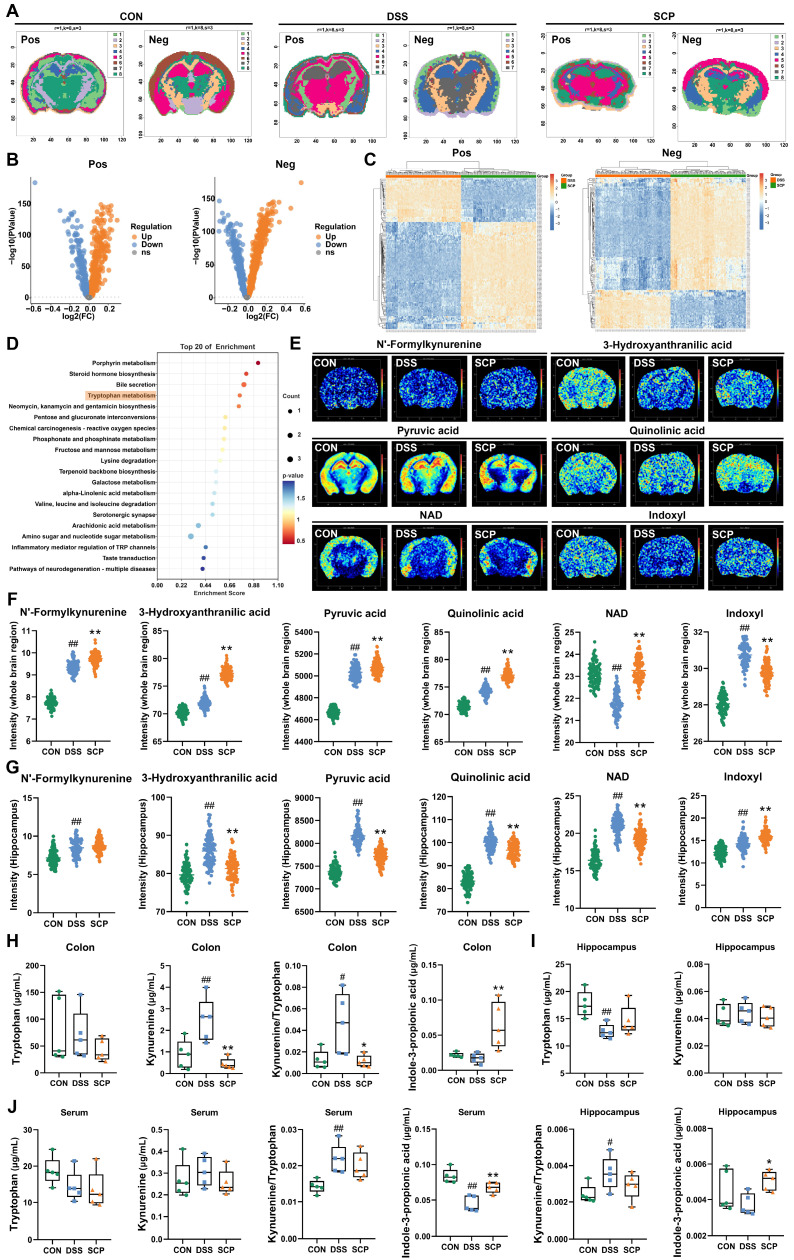
Spatial and targeted metabolomics uncovered gut-brain Trp metabolic reprogramming. A SSCC-based demarcation of mouse brain regions, B Volcano plot of whole-brain differential metabolites (SCP versus DSS), C Hierarchical clustering of differential metabolites in positive/negative ion modes, D KEGG pathway analysis of cerebral Trp metabolism modulated by SCP, E Spatial imaging of Trp derivatives in the mouse brain, F Whole-brain quantification of Trp derivatives, G Hippocampus-specific quantification of Trp derivatives, H Targeted metabolomics analysis of Trp metabolism in colon (n=5/group), I Targeted metabolomics analysis of Trp metabolism in hippocampus (n=5/group), J Targeted metabolomics analysis of Trp metabolism in serum (n=5/group). ^#^*p* < 0.05, ^##^*p* < 0.01 versus CON group; ^*^p < 0.05, ^**^p < 0.01 versus DSS group.

Although SCP treatment rebalanced Trp metabolism in the colon and serum of UC mice, its impact on cerebral Trp metabolism (particularly in specific brain regions) remained unclear. To investigate this, we applied high-resolution (100 μm) spatial metabolomics (AFDESI-MSI) for comprehensive evaluation. Pre-metabolomic HE staining revealed inflammatory infiltration in the hippocampus of UC mice, which was markedly alleviated by SCP intervention (Fig. S3A). Spatial shrunken centroids clustering clearly delineated anatomical boundaries of mouse brain regions (Fig. 5A), aligning with metabolite-based clustering (Fig. S3B). OPLS-DA demonstrated that SCP globally corrected UC-associated cerebral metabolic disturbances (Fig. S3C). In positive ion mode, SCP upregulated 326 metabolites and downregulated 203; in negative ion mode, it upregulated 633 and downregulated 449 metabolites (|FC| > 1, *p* < 0.05) (Fig. 5B). Cluster heatmaps visualized distinct expression patterns of these differential metabolites between SCP and DSS groups (Fig. 5C). Notably, KEGG enrichment analysis confirmed significant SCP-driven remodeling of cerebral Trp metabolism (Fig. 5D). Spatial mapping of Trp derivatives (Fig. 5E) demonstrated preferential accumulation of kyn pathway products (3-hydroxyanthranilic acid, pyruvic acid, QA) in the hippocampus. However, whole-brain quantification indicated that SCP intervention restored only NAD and indoxyl levels (*p* < 0.05), suggesting region-specific modulation of Trp metabolism (Fig. 5F). Given SCP's proven efficacy in mitigating hippocampal neuronal damage and the spatial overlap between metabolite distribution and histopathological changes, we hypothesized hippocampal dominance in SCP-mediated metabolic regulation. To validate this hypothesis, we specifically extracted metabolite data from the hippocampal region (boundaries defined in Fig. S3D). Correlation heatmaps demonstrated a strong linkage between hippocampal Trp metabolism and behavioral parameters (Fig. S3E). Crucially, SCP treatment significantly reduced hippocampal levels of kyn pathway metabolites (N'-formylkynurenine, 3-hydroxyanthranilic acid, pyruvic acid, QA, and NAD) (*p* < 0.05), indicating targeted suppression of hippocampal kyn pathway hyperactivity (Fig. 5G).

To further quantify Trp metabolic alterations along the gut-brain axis, we performed targeted metabolomics analyses of the colon, hippocampus, and serum. OPLS-DA reconfirmed that SCP effectively improved Trp metabolic imbalance along the gut-brain axis (Fig. S3F). In the colon, SCP intervention reduced Kyn levels along with the Kyn/Trp ratio, while significantly increasing IPA levels (Fig. 5H). In the hippocampus, SCP administration restored Trp and IPA levels while markedly decreasing the Kyn/Trp ratio (Fig. 5I). Serum analysis showed SCP markedly increased IPA and decreased Kyn/Trp ratio (Fig. 5J), identifying IPA as a key therapeutic mediator. Considering that IDO1 serves as the key rate-limiting enzyme regulating Trp conversion to Kyn 23, we further examined its activity. RT-PCR and Western blot revealed SCP-mediated suppression of IDO1 mRNA and protein expression in both the colon and hippocampus (Figs. S3G-I). Collectively, these findings indicated that SCP attenuated kyn pathway hyperactivity in the colon, serum, and hippocampus while simultaneously promoting IPA production through IDO1 inhibition.

### SCP alleviated UC and comorbid depression via AhR activation

**Figure 6:**
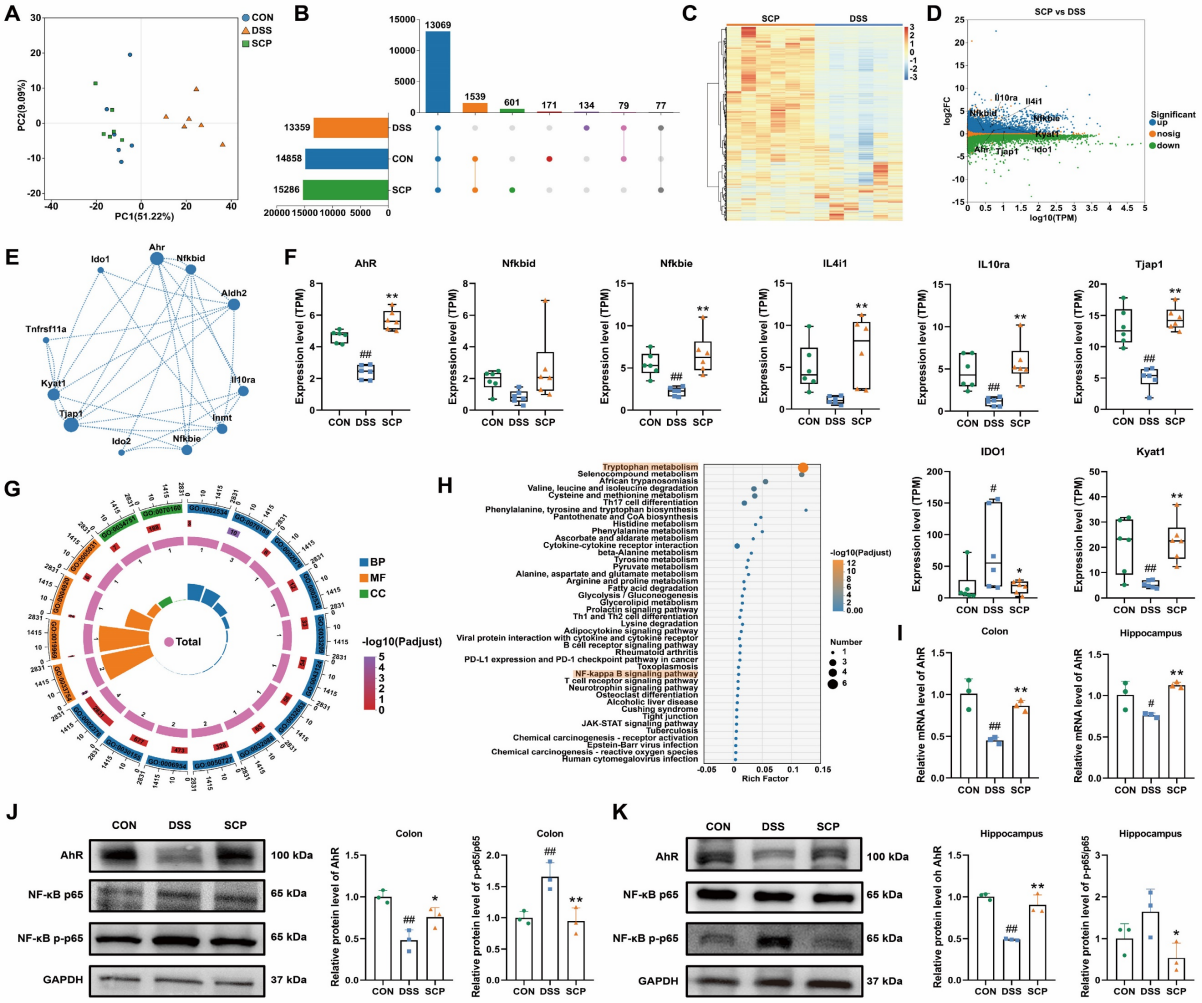
SCP attenuated UC-depression comorbidity via the AhR/NF-κB Pathway. A PCA of colonic transcriptomes, B Number of detected genes, C DEG heatmap (SCP versus DSS), D DEG MA plot (SCP versus DSS). E Spearman correlation network of DEGs. F Quantitative analysis of DEGs (n=6/group). G GO enrichment of DEGs. H KEGG pathway enrichment of DEGs. I AhR mRNA levels in colon and hippocampus (n=3/group). J AhR/NF-κB protein levels in colon (n = 3/group), K AhR/NF-κB protein levels in hippocampus (n = 3/group). ^#^*p* < 0.05, ^##^*p* < 0.01 versus CON group; ^*^p < 0.05, ^**^p < 0.01 versus DSS group.

To identify potential therapeutic targets of SCP in UC and comorbid depression, we analyzed colonic gene expression profiles using RNA-seq. Principal component analysis demonstrated that SCP intervention significantly reversed DSS-induced transcriptional alterations, shifting the profile toward that of the CON group (Fig. 6A). A total of 1539 genes were restored to baseline expression levels by SCP treatment (Fig. 6B). DESeq2 analysis identified differentially expressed genes (DEGs) (|FC| > 1.2, *p* < 0.05), visualized via hierarchical clustering and minus-versus-add (MA) plots. SCP upregulated 3278 genes (e.g., AhR, NFKBID, NFKBIE, IL4I1, IL10RA, TJP1, KYAT1) and downregulated 2779 genes (e.g., IDO1) compared to the DSS group (Fig. 6C, D). Spearman correlation network analysis demonstrated revealed functional associations between AhR and genes including NFKBID, NFKBIE, IL4I1, IL10RA, and KYAT1 (Fig. 6E). Quantitative analysis confirmed SCP-mediated upregulation of AhR, NFKBIE, IL4I1, IL10RA, Tjap1, and KYAT1, alongside IDO1 suppression (*p* < 0.05) (Fig. 6F). GO enrichment analysis highlighted DEG involvement in Kyn metabolic process (GO: 0070189), inflammatory response (GO: 0050727), TNF signaling pathway (GO: 0033209), and cell adhesion (GO: 0030155) (Fig. 6G). KEGG pathway analysis further validated enrichment of Trp metabolism, NF-κB signaling, TJ, and cytokine-cytokine receptor interaction pathways (Fig. 6H). Targeted metabolomics confirmed elevated colonic IPA, a potent AhR ligand, in SCP-treated mice. We proposed that SCP activated AhR via IPA to suppress NF-κB signaling, thereby mitigating intestinal inflammation. Hippocampal IPA elevation suggested parallel AhR activation in the brain. RT-PCR validated SCP-induced AhR mRNA upregulation in both colon and hippocampus (*p* < 0.05) (Fig. 6I), while Western blot confirmed increased AhR protein expression and reduced phosphorylated NF-κB p65 levels in these tissues (*p* < 0.05) (Fig. 6J, K). Collectively, SCP ameliorated UC-depression comorbidity by activating AhR (via IPA) and inhibiting NF-κB p65 phosphorylation across the gut-brain axis.

### SCP-modulated gut microbiota remodeling alleviated UC and comorbid depressive behaviors in mice

**Figure 7:**
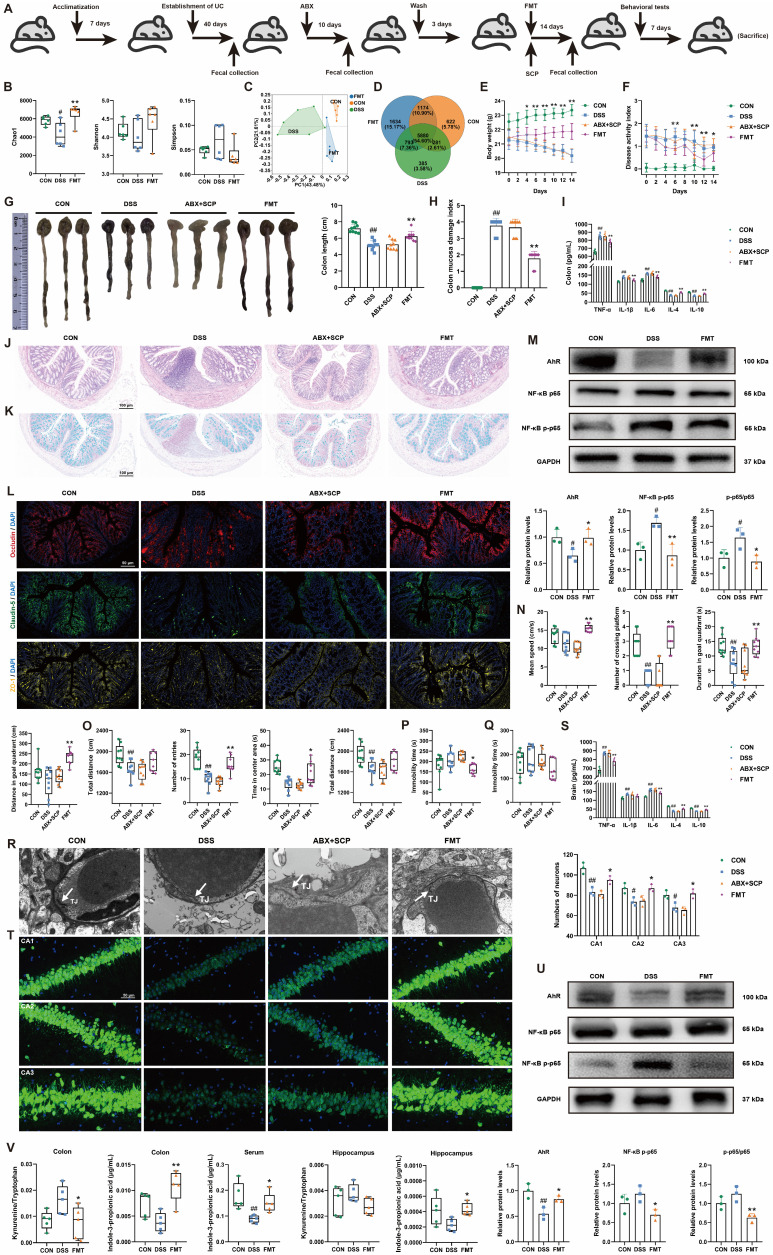
FMT restored gut microbiota and alleviated UC and associated depression. A Schematic of ABX and FMT protocols, B FMT improved α-diversity indices (n=6/group), C PCoA analysis demonstrating FMT-mediated restoration of β-diversity, D Venn diagram illustrating FMT-driven increases in gut microbial species, E Body weight dynamics during ABX/FMT treatment (n=12/group), F DAI scores dynamics during ABX/FMT treatment (n=12/group), G Colon length comparisons across groups (n=9/group), H CMDI scores comparisons across groups (n=9/group), I Colonic inflammatory cytokine levels (n=6/group), J Representative HE-stained colon sections, K Representative Alcian blue-stained colon sections, L Immunofluorescence staining of colonic TJ proteins, M Western blot analysis of AhR/NF-κB p65 pathway in colon (n=3/group), N MWM performance (n=9/group), O OFT parameters (n=9/group), P Immobility time in TST (n=9/group), Q Immobility time in FST (n=9/group), R TEM of BBB ultrastructure, S Brain inflammatory cytokine levels (n=6/group), T Immunofluorescence analysis of hippocampal neuronal damage (n=3/group), U Western blot analysis of hippocampal AhR/NF-κB p65 pathway (n=3/group), V FMT-driven alterations in Trp metabolism across colon, serum, and hippocampus (n=5/group). ^#^*p* < 0.05, ^##^*p* < 0.01 versus CON group; ^*^p < 0.05, ^**^p < 0.01 versus DSS group.

To confirm the necessity of gut microbiota remodeling in mediating SCP's therapeutic effects, we performed ABX-induced microbiota depletion and FMT experiments (Fig. 7A). ABX treatment significantly reduced Chao1 and Shannon indices while elevating the Simpson index (*p* < 0.01), indicating effective eradication of microbial diversity (Fig. S4A). β-Diversity analysis and microbial abundance/composition profiling confirmed near-complete elimination of the original gut microbiota by ABX (Fig. S4B-D). Subsequently, we evaluated the microbial remodeling capacity of FMT using microbiota from SCP-treated donors. FMT intervention reversed DSS-induced α-diversity decline, particularly reflected in the Chao1 index (*p* < 0.05) (Fig. 7B; and Fig. S4E). PCoA analysis further demonstrated that FMT ameliorated DSS-driven structural dysbiosis (Fig. 7C; and Fig. S4F). At the phylum, genus, and species levels, FMT-restored microbial profiles closely resembled those of the SCP-treated group (Fig. S4G), with a notable increase in microbial number in recipient mice (Fig. 7D; and Fig. S4H). These findings indicated successful transfer of SCP-associated beneficial microbiota to recipients.

Regarding anti-UC effects, ABX completely abolished SCP's improvements in body weight, DAI scores, colon length, and CMDI scores (Fig. 7E-H). In contrast, FMT recapitulated the therapeutic benefits of SCP. ELISA showed that ABX negated SCP's regulatory effects on colonic and serum inflammatory cytokines, whereas FMT significantly reversed these abnormalities (*p* < 0.01) (Fig. 7I; and Fig. S4I). HE staining (Fig. 7J; and Fig. S4J) and Alcian blue staining (Fig. 7K; and Fig. S4K) confirmed that ABX+SCP failed to mitigate DSS-induced inflammatory infiltration and goblet cell loss, whereas FMT alleviated these pathological alterations. Immunofluorescence and Western blot revealed that FMT (but not ABX+SCP) upregulated colonic TJ proteins (Occludin, Claudin-5, and ZO-1) expression (Fig. 7L; and Fig. S4L), indicating restored intestinal barrier integrity. Mechanistically, FMT activated colonic AhR signaling and suppressed NF-κB p65 phosphorylation (Fig. 7M). In terms of antidepressant effects, ABX similarly blocked SCP's alleviation of depressive-like behaviors (assessed via MWM, OFT, TST, and FST) in UC mice. Conversely, FMT recipients exhibited significantly improved depressive-like behaviors (Fig. 7N-Q; and Fig. S4M-P). TEM showed that FMT restored BBB TJ integrity, which remained unaltered in the ABX+SCP group (Fig. 7R). Western blot further confirmed FMT-mediated reversal of Occludin and Claudin-5 downregulation in the BBB (*p* < 0.05) (Fig. S4Q). ELISA revealed reduced pro-inflammatory cytokine levels in FMT-treated brains (Fig. 7S). Nissl staining and NeuN^+^ immunofluorescence demonstrated that FMT attenuated hippocampal neuronal damage and increased surviving neuron counts (Fig. 7T; and Fig. S4R). TEM and Western blot further indicated that FMT enhanced hippocampal synaptic plasticity and upregulated PSD-95 expression (Fig. S4S, T). Mechanistically, FMT activated hippocampal AhR signaling and inhibited NF-κB p65 phosphorylation (Fig. 7U). Targeted metabolomics confirmed that FMT significantly reduced the Kyn/Trp ratio in the colon and hippocampus while elevating IPA levels in the colon, serum, and hippocampus (*p* < 0.05) (Fig. 7V). Western blot further validated FMT-mediated suppression of IDO1 overexpression in the colon and hippocampus (*p* < 0.01) (Fig. S4U). In conclusion, FMT-driven gut microbiota recapitulated SCP's effects by rebalancing the gut-brain axis Trp metabolism (inhibiting the kyn pathway and promoting IPA generation), thereby confirming that microbiota remodeling underlies SCP's therapeutic efficacy against UC and associated depressive behaviors.

### IPA alleviated UC and associated depression via AhR activation

**Figure 8:**
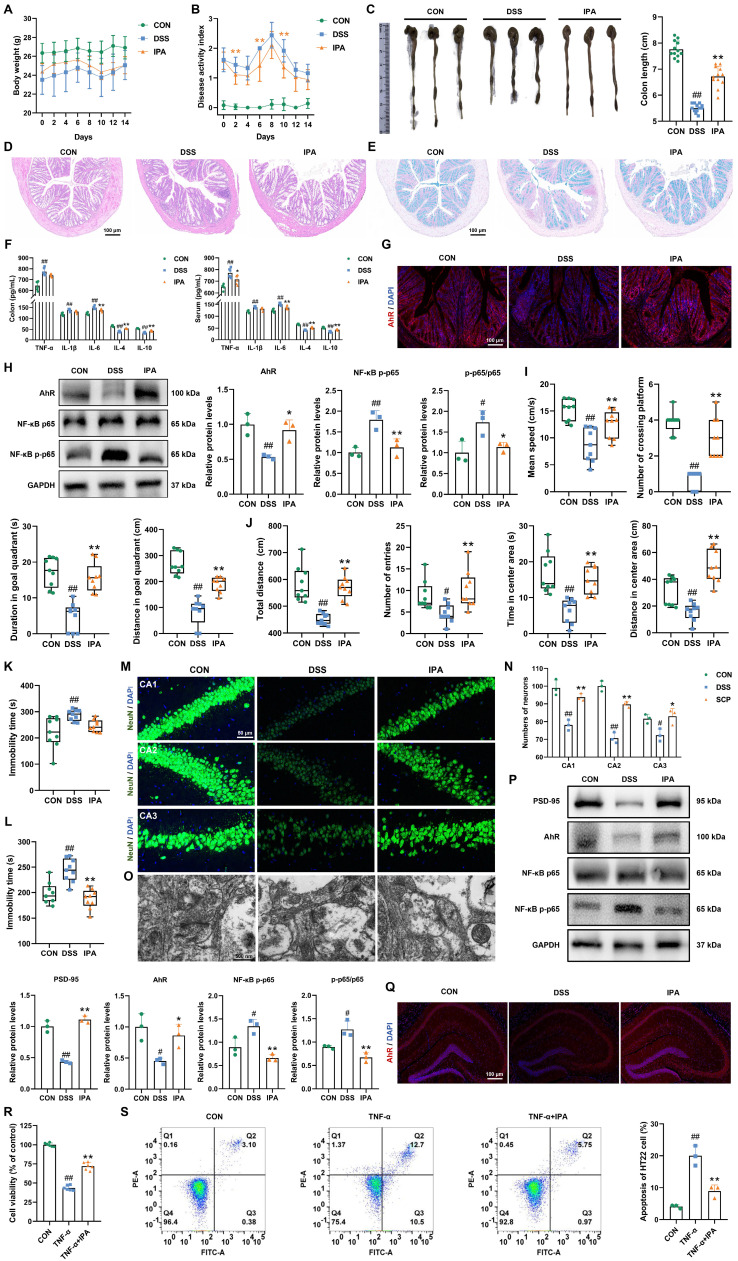
IPA alleviated UC and associated depression through modulation of the AhR/NF-κB signaling pathway. A Restoration of body weight by IPA administration (n=12/group), B Amelioration of DAI scores following IPA treatment (n=12/group), C Colon morphology and quantitative analysis of colon length (n=12/group), D Representative HE-stained colon sections, E Representative Alcian blue-stained colon sections, F ELISA detection of colonic and serum inflammatory cytokine levels (n=6/group), G Immunofluorescence visualization of AhR activation in colonic tissue, H Western blot analysis of AhR and NF-κB p65 phosphorylation in colon (n=3/group), I Behavioral alterations in MWM (n=9/group), J Behavioral alterations in OFT (n=9/group), K Immobility time in the TST (n=9/group), L Immobility time in the FST (n=9/group), M NeuN^+^ immunofluorescence staining of hippocampal neurons, N Quantitative assessment of hippocampal neuronal density (n=3/group), O Synaptic ultrastructure revealed by TEM imaging, P Hippocampal protein expression of AhR, NF-κB p-p65, and PSD-95 (n=3/group), Q Immunofluorescence visualization of AhR activation in hippocampal tissue, R Neuronal viability measured by CCK-8 assay (n=6/group), S Flow cytometry quantification of neuronal apoptosis rates (n=3/group). ^#^*p* < 0.05, ^##^*p* < 0.01 versus CON group; ^*^p < 0.05, ^**^p < 0.01 versus DSS group.

Previous multi-omics and FMT experiments suggested that IPA is a key metabolite mediating the therapeutic effects of SCP. To directly validate IPA's efficacy, we conducted IPA supplementation experiments in UC mice (Fig. S5A). IPA intervention effectively ameliorated DSS-induced body weight loss (Fig. 8A), elevated DAI scores (Fig. 8B), colon shortening (Fig. 8C), and mucosal damage (Fig. S5B). HE and Alcian blue staining revealed that IPA significantly reduced inflammatory cell infiltration and goblet cell loss in colon tissues (Fig. 8D, E; and Fig. S5C, D), promoting structural restoration. ELISA showed IPA markedly decreased pro-inflammatory cytokine levels while increasing anti-inflammatory cytokine levels in both colon and serum (*p* < 0.05), indicating alleviation of intestinal and systemic inflammation (Fig. 8F). Western blot confirmed IPA upregulated TJ proteins expression in the intestinal barrier (*p* < 0.05), suggesting reduced permeability (Fig. S5E). To determine whether IPA's therapeutic effects depend on AhR, we assessed its expression. Immunofluorescence demonstrated that IPA significantly enhanced AhR fluorescence intensity (Fig. 8G). Western blot confirmed IPA increased AhR protein levels while reducing NF-κB p65 phosphorylation (*p* < 0.05) (Fig. 8H), indicating IPA mitigated intestinal inflammation via AhR activation. Behaviorally, IPA supplementation replicated SCP's antidepressant effects by improving performance of UC mice in the MWM, OFT, TST, and FST (Fig. 8I-L; and Fig. S5F-I). TEM and Western blot analyses demonstrated IPA enhanced BBB TJs (Fig. S5J, K). Nissl staining (Fig. S5L, M) and NeuN^+^ immunofluorescence (Fig. 8M, N) consistently confirmed that IPA preserved hippocampal neurons and increased neuronal survival (*p* < 0.05). TEM revealed IPA improved synaptic structure by increasing postsynaptic density length and width while reducing synaptic cleft, and Western blot confirmed the restoration of PSD-95 expression (*p* < 0.01) (Fig. 8O, P). Further analyses indicated that IPA upregulated hippocampal AhR expression, suppressed NF-κB p65 phosphorylation (*p* < 0.05) (Fig. 8P, Q), and normalized brain cytokine levels (Fig. S5N). Critically, fluorescence imaging of FITC-IPA in brain tissue sections clearly demonstrated its ability to cross the BBB, providing direct evidence for its gut-brain axis trafficking (Fig. S5O).

*In vitro*, IPA demonstrated protective effects against TNF-α induced inflammatory damage in mouse hippocampal neurons. CCK-8 assays confirmed that IPA was non-toxic at concentrations up to 100 μmol/L (Fig. S5P). At a concentration of 5 μmol/L, IPA significantly alleviated TNF-α induced neuronal injury (*p* < 0.01) (Fig. 8R). Flow cytometry showed that IPA reduced apoptosis (*p* < 0.01) (Fig. 8S), and ELISA confirmed a decrease in pro-inflammatory cytokine levels in HT22 cells (Fig. S5Q). In summary, IPA recapitulated SCP's core therapeutic benefits against UC and comorbid depression by activating the gut-brain AhR pathway and suppressing NF-κB-mediated inflammation.

### AhR is the critical target for SCP in ameliorating UC and associated depression

**Figure 9:**
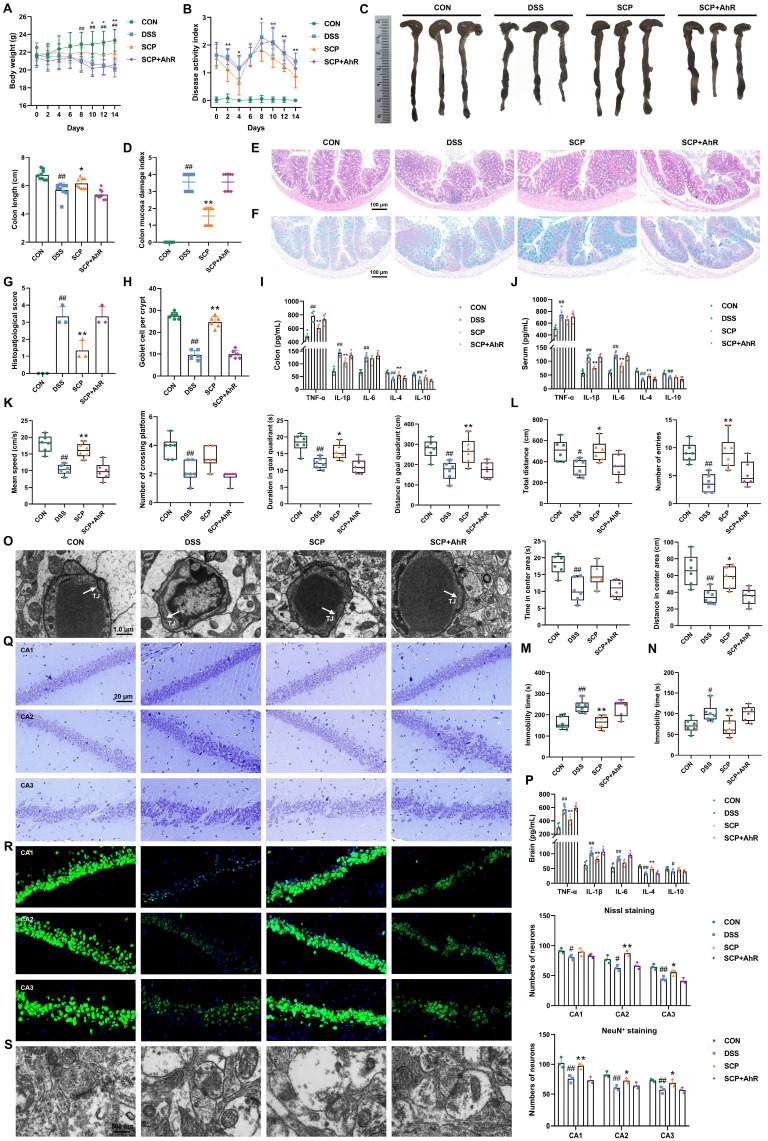
AhR served as the pivotal mediator of SCP's therapeutic effects against UC-depression comorbidity. A Body weight dynamics across groups (n=12/group), B DAI score progression (n=12/group), C Representative colon images with length quantification (n=9/group), D CMDI evaluation (n=9/group), E Representative HE-stained colon sections, F Representative Alcian blue-stained colon sections, G Histopathological scores of colon tissue (n=3/group), H Goblet cell density quantification (n=6/group), I Inflammatory cytokine levels in colon (n=6/group), J Inflammatory cytokine levels in serum (n=6/group), K Cognitive performance metrics in MWM (n=6/group), L Locomotor and exploratory behaviors in OFT (n=6/group), M Immobility in the TST (n=6/group), N Immobility in the FST (n=6/group), O Ultrastructural integrity of the BBB, P Neuroinflammatory cytokine levels in brain (n=6/group), Q Hippocampal neuronal architecture visualized by Nissl staining (n=3/group), R Neuronal survival assessment via NeuN^+^ immunofluorescence (n=3/group), S Synaptic ultrastructure revealed by TEM imaging. ^#^*p* < 0.05, ^##^*p* < 0.01 versus CON group; ^*^p < 0.05, ^**^p < 0.01 versus DSS group.

Previous findings demonstrated that SCP alleviated UC comorbidity by rebalancing Trp metabolism and enhancing IPA-mediated AhR activation. To confirm AhR as the critical therapeutic target, DSS-treated mice were co-administered an AhR inhibitor in combination with SCP. The AhR inhibitor completely abolished SCP-mediated improvements in UC symptoms, including body weight recovery, DAI and CMDI scores, colon length, inflammatory infiltration, goblet cell depletion, and dysregulated colonic and serum cytokine levels (TNF-α, IL-1β, IL-6, IL-4, and IL-10) (Fig. 9A-J). Concurrently, the antidepressant effects of SCP were fully reversed by AhR inhibition, as evidenced by impaired performance in behavioral tests (MWM, OFT, TST, and FST), increased BBB permeability, elevated cerebral inflammatory cytokines, reduced hippocampal neuronal activity, and impaired synaptic plasticity (Fig. 9K-S).

## Discussion

In this study, we demonstrated that DSS-induced UC mice triggered intestinal inflammation and compromised intestinal barrier integrity, permitting uncontrolled systemic dissemination of pro-inflammatory cytokines. The cumulative inflammatory mediators in circulation subsequently impaired TJ structures of the BBB, facilitating neuroinflammatory infiltration into the CNS. Progressive neuroinflammation exacerbated hippocampal neuronal damage and synaptic plasticity impairment. SCP remodeled the gut microbiota in UC mice, restoring intestinal barrier integrity through upregulation of TJ proteins and mucin production, thereby effectively attenuating systemic dissemination of pro-inflammatory cytokines. Crucially, SCP established a direct gut-brain connection by profoundly reprogramming microbial Trp metabolism from the IDO1-dominated neurotoxic Kyn pathway towards IPA biosynthesis.

**Figure 10:**
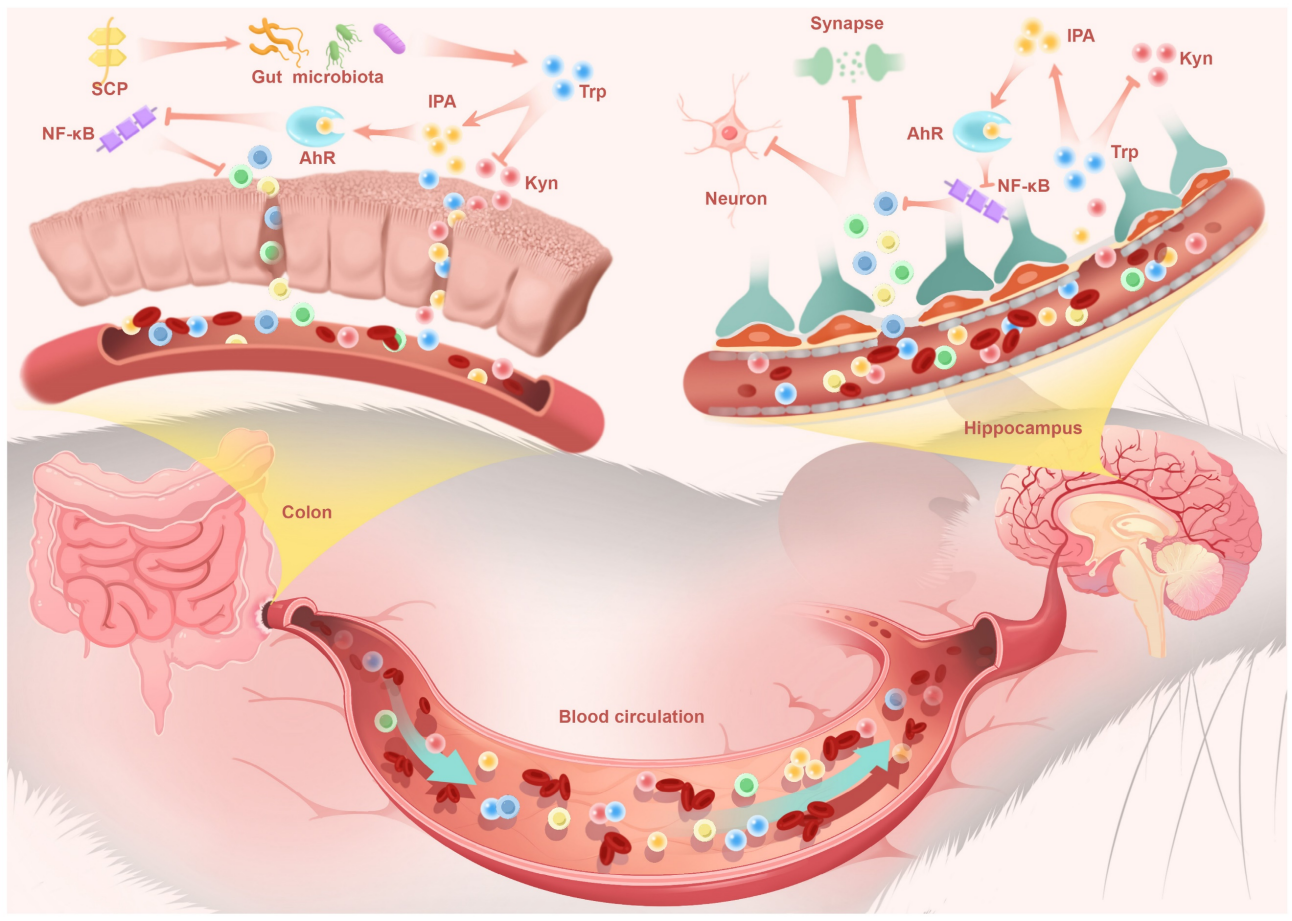
SCP reshaped the gut microbiota to drive a rebalancing of its mediated Trp metabolism, thereby increasing IPA levels in the colon, serum, and hippocampus. IPA alleviated colonic and neuroinflammation by activating the downstream AhR and inhibiting the NF-κB signaling pathway, while protecting hippocampal neurons and synapses. Additionally, SP restored the integrity of the intestinal barrier and BBB, mitigating disruptions in gut-brain signaling communication.

This metabolic shift proved pivotal as IPA, a potent AhR agonist, entered systemic circulation. Functioning as a key gut-derived messenger, circulating IPA executed CNS effects via the gut-brain axis. Upon reaching the hippocampus, IPA activated neuronal AhR signaling, which subsequently suppressed NF-κB-mediated neuroinflammation. This direct central mechanism underpinned the observed neuroprotective effects: preservation of hippocampal neurons, restoration of synaptic plasticity, and enhanced BBB integrity, collectively reversing depressive behaviors in UC mice (Fig. 10). These findings not only advanced our understanding of the pathophysiological interplay underlying UC-associated neuropsychiatric disorders but also identified SCP as a promising therapeutic candidate targeting the microbiome-gut-brain axis for clinical translation.

*Schisandra chinensis* (Turcz.) Baill., a pharmacopeial medicinal plant documented in China, Korea, and Japan, produces mature fruits traditionally utilized in East and Northeast Asian ethnomedicine. Polysaccharides derived from SC represent pharmaceutically valuable natural products with established safety profiles and multifunctional therapeutic potential. Emerging evidence demonstrated that SC polysaccharide extracts exerted neuroprotective effects through mechanisms involving anti-inflammatory cytokine modulation and hippocampal neuron repair, thereby ameliorating neuropsychiatric disorders including depression, cognitive impairment, and Parkinson's disease 24-26. Our prior research, corroborated by independent studies, further revealed their capacity to restore gut microbiota homeostasis and rectify microbial metabolite imbalances in UC models 16, 27. Based on these findings, we isolated and purified a novel low-molecular-weight polysaccharide from SC and investigated its therapeutic effects and mechanisms against UC with comorbid depression. Although the precise etiology linking UC to depression remains unclear, dysbiosis of the gut microbiota and its metabolites undoubtedly plays a pivotal role 28, 29. Specifically, UC leads to a reduction in beneficial bacteria and an increase in pathogenic bacteria within the gut, disrupting the synthesis or degradation processes of their associated metabolites. These alterations subsequently impact CNS function via transmission along the microbiota-gut-brain axis. Studies have indicated that low-molecular-weight polysaccharides are more readily utilized by the gut microbiota, promoting the proliferation of probiotics, which in turn modulates intestinal barrier function and inflammatory responses. Moreover, α-glucans exhibit favorable biocompatibility and biodegradability, contributing to the restoration of intestinal homeostasis. The abundant α-(1→4) glycosidic bonds in the main chain of SCP, along with the branched structures, provide ample substrates for microbial glycoside hydrolases 30, 31. SCP significantly increased the abundance of beneficial bacteria such as *Dubosiella muris*, *Lactobacillus taiwanensis*, *Limosilactobacillus reuteri*, and *Allobaculum mucilyticum*, which have been shown to be closely associated with UC and depression indices. ABX depletion and FMT experiments further confirmed the necessity of the gut microbiota for SCP's therapeutic effects. Integrated KEGG analysis of metagenomics and untargeted metabolomics consistently identified gut microbiota-regulated Trp metabolism as the primary pathway through which SCP ameliorated UC and associated depression.

Trp, an essential amino acid, is produced by the gut microbiota through dietary fiber breakdown. Trp and its downstream derivatives form a complex metabolic network, maintaining a competitive balance between its three major metabolic pathways under normal physiological conditions 32. During UC, IDO1 activity is strongly induced, leading to excessive accumulation of the Kyn pathway. For instance, increased kynurenic acid/Trp ratios have been detected in the serum of UC patients, correlating positively with endoscopic sub-scores 33. Moreover, since Trp and most of its metabolites can cross the BBB and enter the CNS, neurological function is profoundly influenced by Trp metabolic balance. Metabolites along the Kyn pathway, such as 3-hydroxykynurenine and QA, are well-known neurotoxins. Their accumulation in the brain from the bloodstream generates local free radicals, resulting in neuronal degeneration and apoptosis 34, 35. Targeted metabolomics combined with FMT revealed that SCP supplementation suppressed the overactivation of the Kyn pathway in the colon, serum, and hippocampus of UC mice in a gut microbiota-dependent manner, primarily by reducing Kyn levels, the Kyn/Trp ratio, and IDO1 activity. Spatial metabolomics analysis further demonstrated that SCP intervention significantly lowered hippocampal QA levels in UC mice.

The indole pathway, another branch of Trp metabolism, includes indole, indole-3-acetic acid, indole-3-acrylic acid, skatole, IPA, indole-3-aldehyde, among others. Although this pathway accounts for only approximately 5% of total Trp flux, it exhibits potent and broad anti-inflammatory activity 36-38. Our findings showed that SCP intervention significantly elevated IPA levels both within and outside the CNS of UC mice, a process confirmed to be mediated by the gut microbiota via FMT. Notably, as the primary site of IPA production, the colon exhibited lower concentrations of IPA compared to serum. This may be attributed to its rapid absorption into the bloodstream, as well as its potential further degradation or local cellular uptake within the colon 39, 40. IPA, characterized by an aromatic indole ring linked to a propionic acid side chain, has emerged in recent studies as a bioactive molecule with beneficial regulatory effects in both gastrointestinal and neurological disorders. For intestinal protection, IPA significantly reduces intestinal epithelial paracellular permeability, upregulates the expression of TJ proteins, and enhances the mucus barrier by increasing mucins and goblet cell secretions, thereby attenuating LPS-induced intestinal barrier damage 41. Regarding neuroprotection, researchers have found that IPA supplementation reduces the expression of Ccl2 (a pro-inflammatory cytokine) in astrocytes of antibiotic-treated mice, indicating its role in alleviating astrocyte-mediated neuroinflammation 42. By treating UC mice with IPA, we observed that IPA recapitulated the therapeutic effects of SCP, improving colonic inflammation, intestinal barrier function, BBB integrity, neuroinflammation, and hippocampal neuronal and synaptic damage. *In vitro*, IPA demonstrated significant efficacy in protecting against LPS-induced apoptosis in hippocampal neurons. Indole derivatives are established ligands for AhR, a crucial regulator of gut and neuroinflammatory homeostasis and a key initiator of downstream signaling pathways 43. Although the concentration of IPA in the hippocampus was relatively low due to the limitations imposed by the BBB, its high affinity for the AhR still enabled it to exert anti-inflammatory effects 44. Transcriptomic analysis revealed that SCP intervention significantly upregulated AhR gene expression in colonic tissue. Subsequent RT-PCR and Western blot analyses confirmed that SCP significantly increased AhR mRNA and protein expression levels in both the colon and hippocampus. This evidence supported the central role of AhR in modulating intestinal and neuroinflammation. Furthermore, SCP treatment upregulated the expression of genes such as NFKBIE and IL4I1, which have been previously implicated in the regulation of the AhR/NF-κB signaling pathway 45, 46. Western blot analysis confirmed that SCP treatment significantly inhibited the phosphorylation of NF-κB p65. Critically, both FMT and IPA supplementation replicated the therapeutic effects of SCP by activating the AhR pathway, while an AhR inhibitor completely abolished SCP's efficacy. Integrating this evidence, we concluded that SCP regulated inflammatory cytokine levels and alleviates gut-brain axis inflammation via the AhR/NF-κB signaling pathway.

Although we have characterized the structural composition of SCP and demonstrated its efficacy against UC and comorbid depression via the "gut microbiota-Trp metabolism-AhR" axis, certain limitations remain. While SCP is identified as a low-molecular-weight homogeneous polysaccharide, the core structural features responsible for its therapeutic effects and the mechanisms by which it is utilized by gut microbes are not yet fully elucidated. Additionally, while Trp metabolism is a significant gut-brain signaling pathway, the potential involvement of other pathways, such as the gut vagus nerve and the hypothalamic-pituitary-adrenal axis, in SCP's therapeutic process requires further validation. Addressing these aspects is crucial for a comprehensive understanding of SCP's mechanism against UC and associated depression and for advancing its development as a therapeutic agent.

## Supplementary Material

Supplementary figures and tables.

## Data Availability

The authors declare that all data supporting the results of this study are available within the paper and its Supplementary Information. The metagenomic data generated in this study have been deposited in the SRA database under the accession number PRJNA1228347, PRJNA1232368 and PRJNA1233777.

## Abbreviations

5-ASA: mesalazine
5-HT: serotonin
ABX: antibiotic cocktail
AhR: aryl hydrocarbon receptor
BBB: blood-brain barrier
CMDI: colonic mucosal damage index
CNS: central nervous systems
CON: control
DAI: disease activity index
DEG: differentially expressed gene
DSS: dextran sodium sulfate
ELISA: enzyme-linked immunosorbent assay
FITC: fluorescein isothiocyanate
FMT: fecal microbiota transplantation
FST: forced swim test
GO: gene ontology
HE: hematoxylin and eosin
HPGPC: high performance gel permeation chromatography
IBD: inflammatory bowel disease
IDO1: indoleamine 2,3-dioxygenase 1
IPA: indole-3-propionic acid
KEGG: kyoto encyclopedia of genes and genomes
Kyn: kynurenine
LDA: linear discriminant analysis
LEfSe: linear discriminant analysis effect size
LPS: lipopolysaccharide
MA: minus-versus-add
MWM: morris water maze
OFT: open field test
OPLS-DA: orthogonal partial least squares discriminant analysis
PSD-95: postsynaptic scaffolding protein 95
QA: quinolinic acid
SASP: sulfasalazine
SC: *schisandra chinensis*
SCP: *schisandra chinensis* polysaccharide
SCPH: high-dose SCP
SCPL: low-dose SCP
SYN: synaptophysin
TEM: transmission electron microscopy
TJ: tight junction
Trp: tryptophan
TST: tail suspension test
UC: ulcerative colitis
