# Supplementary material for: Gut Microbiota-driven Tryptophan Metabolism Towards the Indole Pathway Mediates Schisandra Chinensis Polysaccharide's Alleviation of Ulcerative Colitis and Comorbid Depression via Aryl Hydrocarbon Receptor
Source: Int J Biol Sci. 2026 Feb 11;22(5):2557–80. doi: 10.7150/ijbs.125012 (PMC12965246; doi:10.7150/ijbs.125012)
Supplement: Supplementary file 1 — Supplementary figures and tables. [file ijbsv22p2557s1.pdf]

Table S1 Sequences of the gene-specific primers.

Gene	Forward primer (5'-3')	Reverse primer (5'-3')
AhR	GAGGTGGGTCCAGTCCAATG	GTCAGTGGTCTCTGAGTGGC
IDO1	CCAGTGCAGTAGAGCGTCAA	TCTGGGTCCACAAAGTCACG
GAPDH	TGCACCACCAACTGCTTAG	AGAGGCAGGGATGATGTTC

Table S2 Methylation analysis of SCP.

PMAA	RT (min)	Molar ratio (%)	Linkage types
1,5-di-O-acetyl-6-deoxy-2,3,4-tri-O-methyl rhamnitol	12.753	1.254	t-Rha*p*
1,2,5-tri-*O*-acetyl-3,4-di-*O*-methyl xylitol	15.643	1.233	1,2-Xyl*p*
1,5-di-*O*-acetyl-2,3,4,6-tetra-*O*-methyl glucitol	16.805	31.216	t-Glc*p*
1,5-di-*O*-acetyl-2,3,4,6-tetra-*O*-methyl galactitol	17.490	4.843	t-Gal*p*
1,4,5-tri-*O*-acetyl-2,3,6-tri-*O*-methyl galactitol	19.809	0.652	1,4-Gal*p*
1,4,5-tri-O-acetyl-2,3,6-tri-O-methyl glucitol	20.062	50.107	1,4-Glc*p*
1,5,6-tri-*O*-acetyl-2,3,4-tri-*O*-methyl glucitol	20.526	3.636	1,6-Glc*p*
1,5,6-tri-*O*-acetyl-2,3,4-tri-*O*-methyl galactitol	21.537	1.479	1,6-Gal*p*
1,4,5,6-tetra-*O*-acetyl-2,3-di-*O*-methyl glucitol	23.319	5.581	1,4,6-Glc*p*

Table S3 ^{1}H and ^{13}C chemical shifts of SCP.

Glycosyl residues			Chemical shift δ (ppm)					
			1	2	3	4	5	6
A	→4)-α-D-Glc*p*-(1→	H	5.30	3.53	3.87	3.56	3.75	3.67/3.76
		C	99.51	71.32	72.99	76.75	70.94	60.41
B	→4,6)-α-D-Glc*p*-(1→	H	5.26	3.48	3.87	3.53	3.75	3.83/3.87
		C	100.13	71.32	72.99	76.74	70.94	66.51
C	→6)-α-D-Glc*p*-(1→	H	4.88	3.45	3.92	3.38	3.84	3.85
		C	98.50	71.19	73.37	69.42	70.05	66.12
D	α-D-Glc*p*-(1→	H	4.86	3.45	3.59	3.31	3.66	3.67/3.76
		C	97.99	71.29	72.61	69.28	72.71	60.41
R_{α}	→4)-α-D-Glc*p*	H	5.13	3.46	3.84	3.57	3.86	3.73/3.82
		C	91.88	71.32	70.18	76.91	73.09	60.41
R_{β}	→4)-β-D-Glc*p*	H	4.55	3.16	3.67	3.53	3.51	3.74/3.80
		C	95.69	73.86	76.04	76.80	74.38	60.54
E	→4)-β-D-Gal*p*-(1→	H	4.54	3.59	3.68	4.08	3.61	3.63/3.71
		C	104.35	71.57	73.06	77.43	74.62	60.40

F	β-D-Gal*p*-(1→	H	4.46	3.52	3.57	3.84	3.70	3.63/3.71
		C	102.42	70.04	72.71	68.66	75.76	60.40
G	→6)-β-D-Gal*p*-(1→	H	4.34	3.43	3.56	3.84	3.78	3.83/3.87
		C	103.18	71.32	72.71	68.66	72.85	69.04

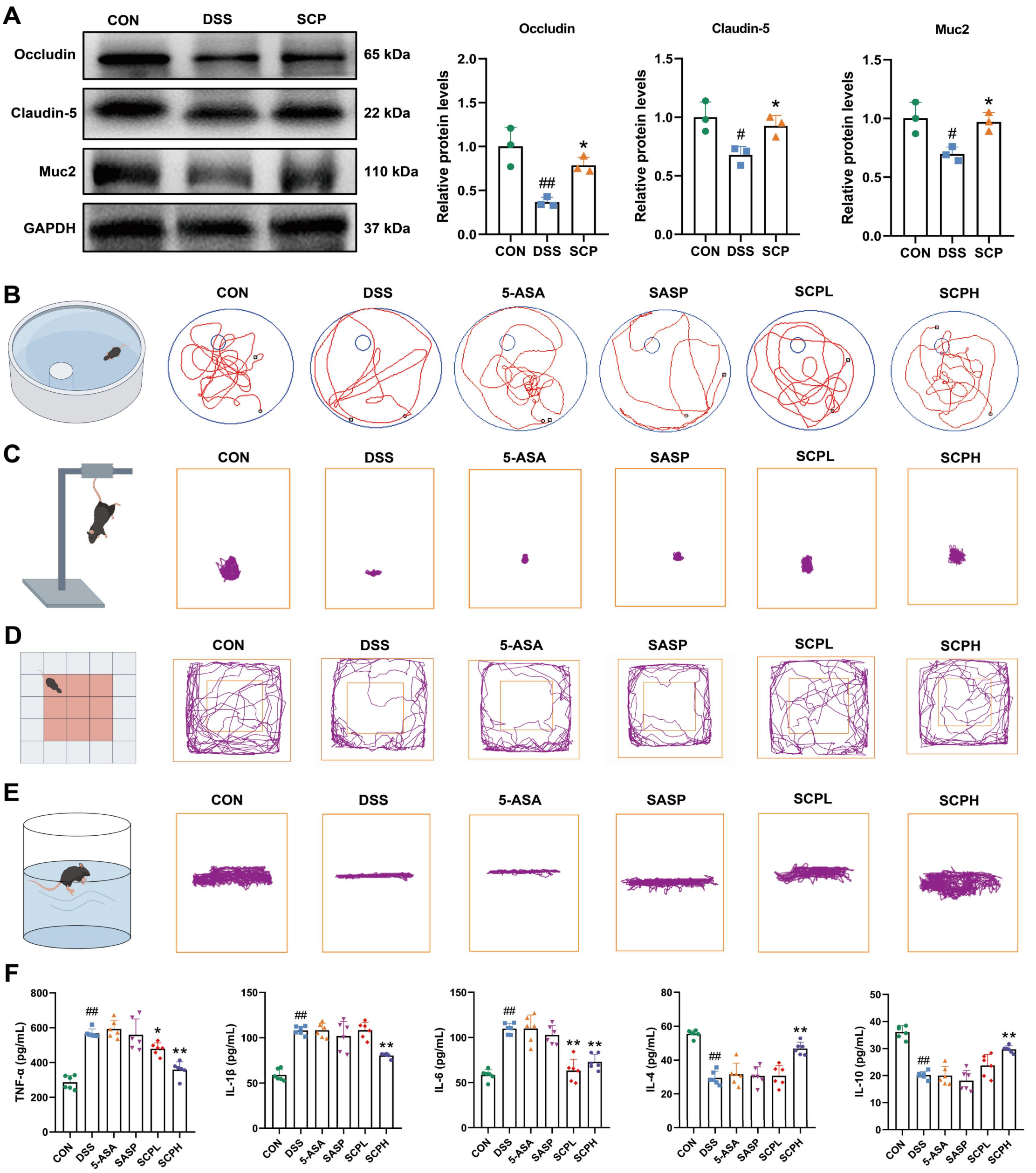

Fig. S1 SCP ameliorated UC and associated depression. A Western blot analysis of TJ proteins (Occludin and Claudin-5) expression in the intestinal barrier (n=3/group), B Swimming trajectories in the MWM, C Struggling trajectories in the TST, D Locomotion tracks in the OFT, E Swimming trajectories in the FST, F ELISA quantification of brain inflammatory cytokines (n=6/group). $^{\#}P < 0.05$, $^{\#\#}P < 0.01$ versus CON group; $^{*}P < 0.05$, $^{**}P < 0.01$ versus DSS group.

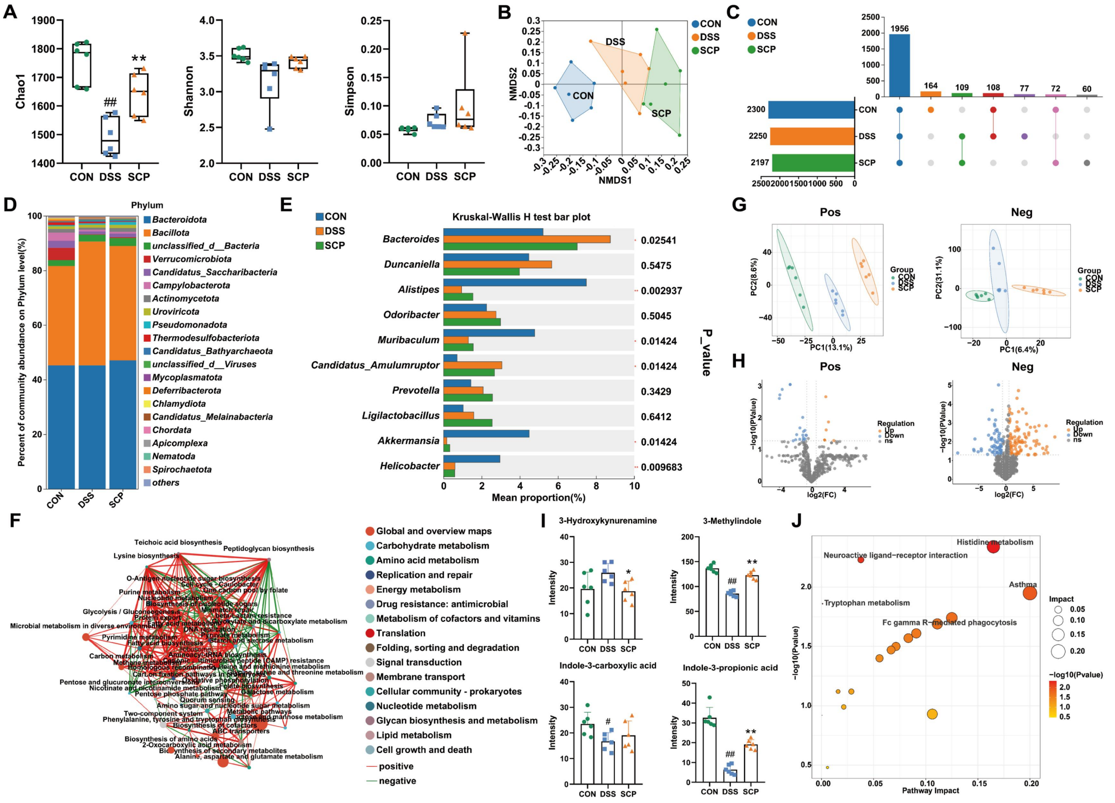

Fig. S2 SCP reshaped gut microbiota and restored serum metabolic homeostasis in UC mice. A α-Diversity analysis at the genus level (n=6/group), B β-Diversity analysis at the genus level, C Number of detected gut microbial taxa (genus), D Phylum-level relative abundance of gut microbiota, E Top 10 differentially abundant genera, F Correlation network of microbial composition, G OPLS-DA score plot of serum metabolomics, H Volcano plot of serum differential metabolites (SCP versus DSS), I Comparison of Trp and its derivatives in serum (n=6/group), J KEGG pathway enrichment analysis of Trp metabolism. $^{\#}P < 0.05$, $^{\#\#}P < 0.01$ versus CON group; $^{*}P < 0.05$, $^{**}P < 0.01$ versus DSS group.

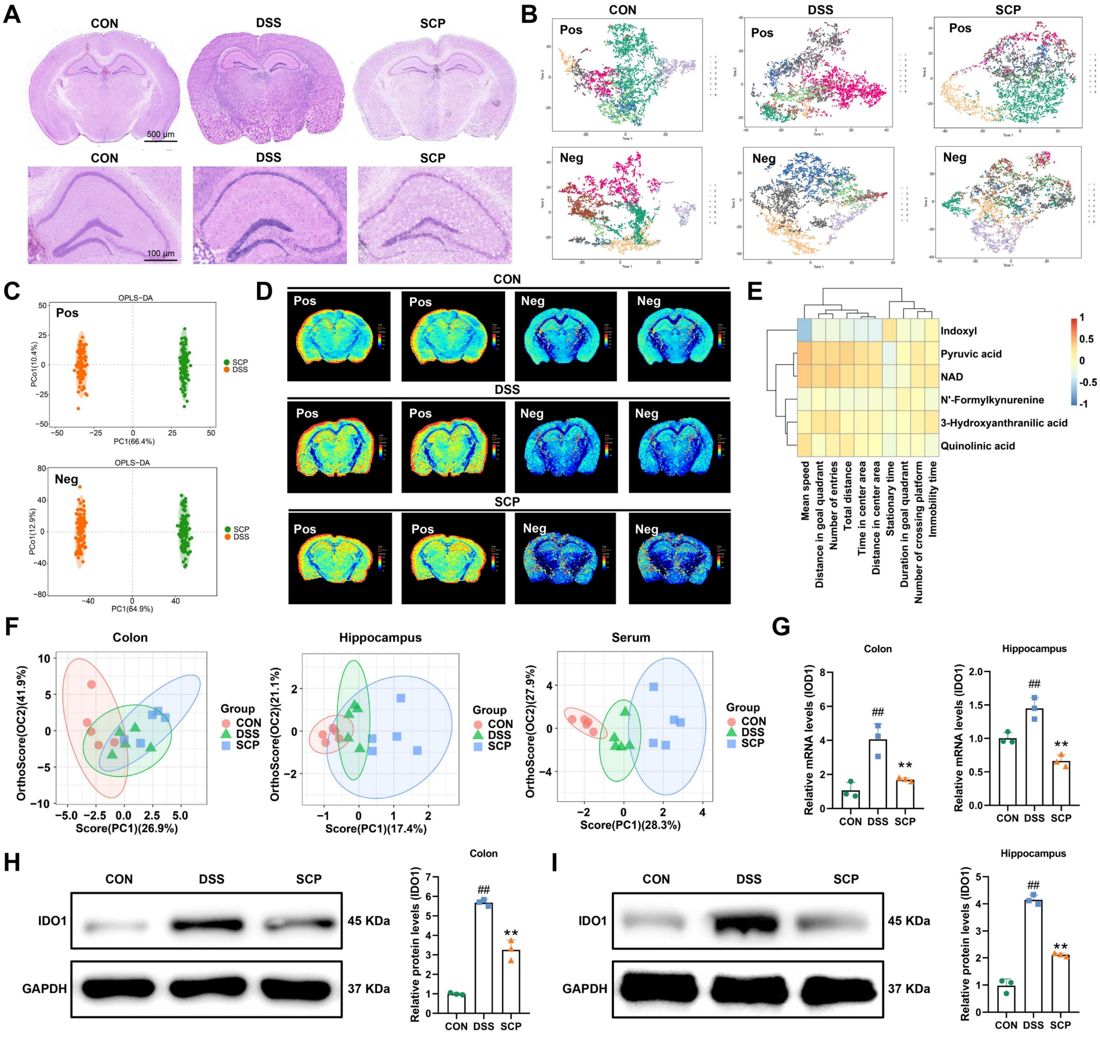

Fig. S3 SCP suppressed Kyn pathway overactivation and promoted IPA production. A HE staining of brain tissue, B Spatial shrunken centroids clustering analysis, C OPLS-DA score plot of whole-brain metabolomics, D Hippocampal subregion demarcation, E Correlation heatmap between Trp derivatives and behavioral parameters, F OPLS-DA score plot of targeted metabolomics, G mRNA expression of IDO1 in colon and hippocampus (n=3/group), H Western blot analysis of colonic IDO1 protein (n=3/group), I Western blot analysis of hippocampal IDO1 protein (n=3/group). $^{##}P < 0.01$ versus CON group; $^{**}P < 0.01$ versus DSS group.

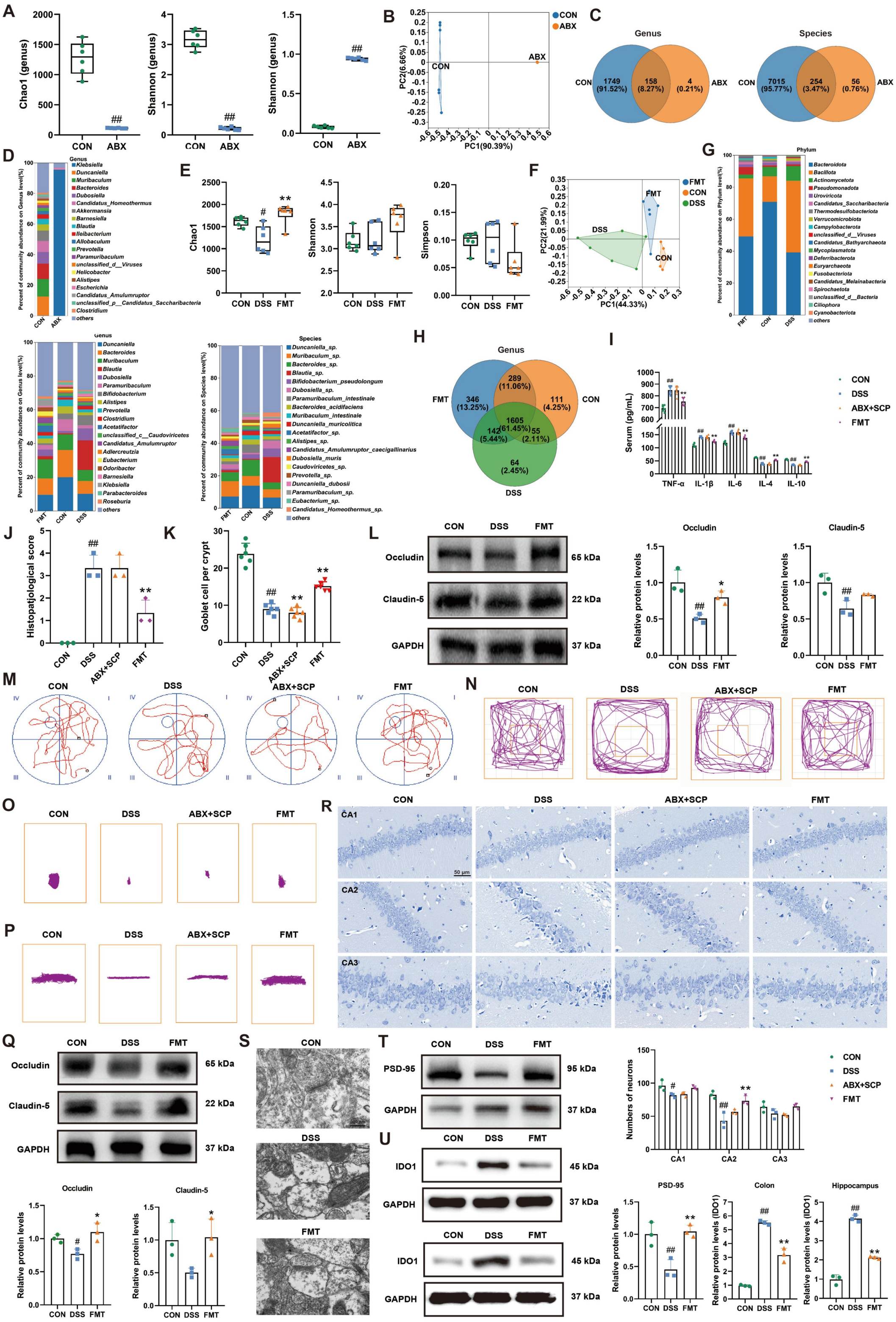

Fig. S4 FMT restored gut microbiota and alleviated UC and associated depression. A α-Diversity changes after ABX treatment (n=6/group), B β-Diversity changes after ABX treatment, C Microbial taxa depletion by ABX, D Phylum-level microbial abundance altered by ABX, E FMT-mediated α-diversity recovery at genus level (n=6/group), F FMT-restored β-diversity at

genus level, G Taxon-specific abundance changes after FMT across phylum, genus, and species levels, H Venn diagram of genus-level taxa increased by FMT, I Serum inflammatory cytokines by ELISA (n=6/group), J Histopathological scoring of colon via HE staining (n=3/group), K Goblet cell quantification (n=6/group), L Western blot of intestinal TJ proteins (n=3/group), M MWM swimming trajectories, N OFT locomotion tracks, O TST struggle patterns, P FST activity traces, Q Western blot of BBB TJs (n=3/group), R Nissl staining of hippocampal neurons, S TEM analysis of synaptic plasticity, T Western blot of PSD-95 expression (n=3/group), U Western blot of IDO1 modulation by FMT (n=3/group). $^{\#}P < 0.05$, $^{\#\#}P < 0.01$ versus CON group; $^{*}P < 0.05$, $^{**}P < 0.01$ versus DSS group.

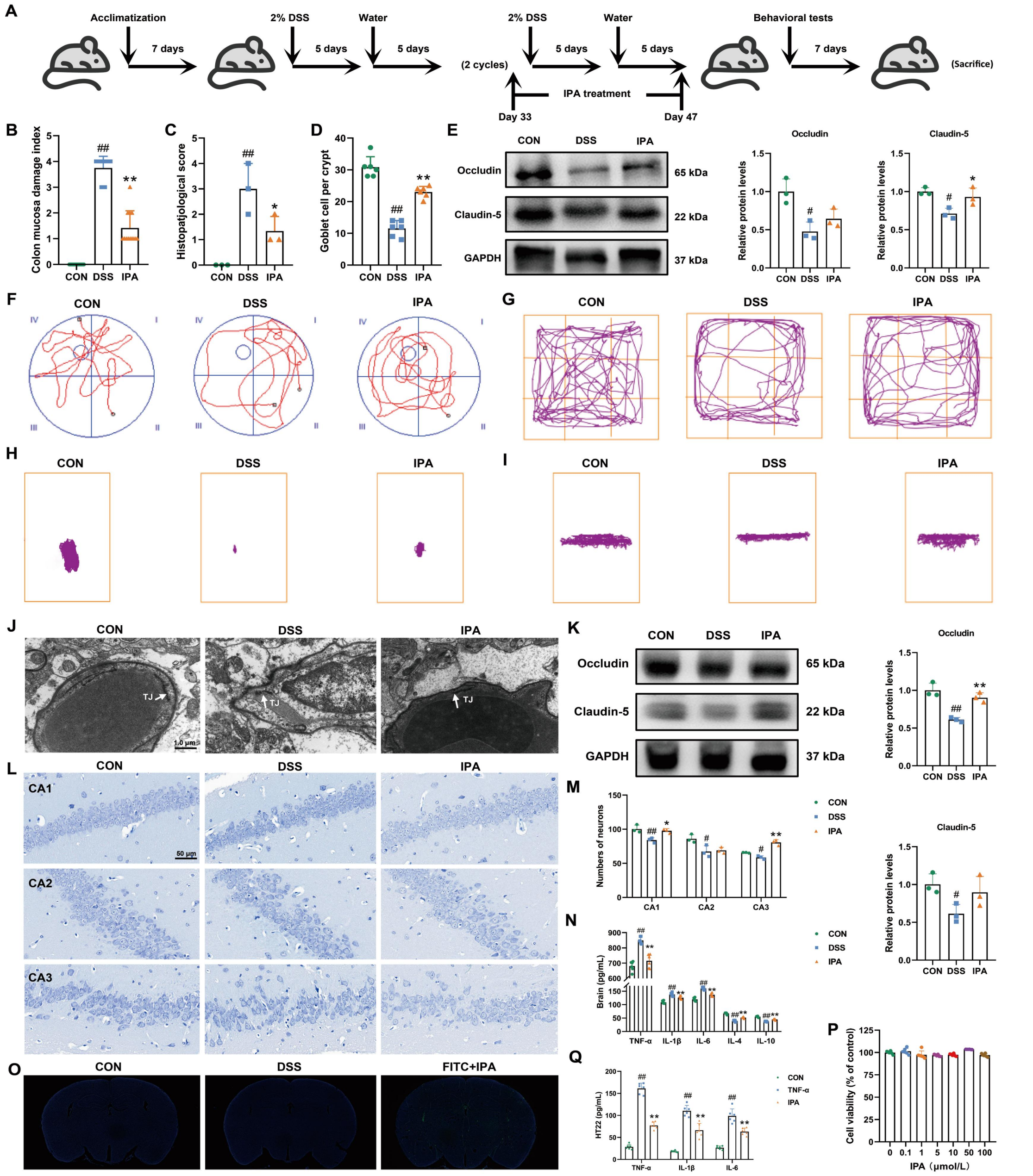

< 0.01 versus DSS group.

Fig. S5 IPA treatment alleviated UC and comorbid depression. A IPA administration protocol, B CMDI scores (n=12/group), C Histopathological scores (n=3/group), D Goblet cell counts (n=6/group), E Western blot of intestinal barrier proteins (n=3/group), F MWM swimming trajectories, G OFT locomotion tracks, H TST struggle patterns, I FST activity traces, J TEM evaluation of BBB ultrastructure, K Western blot of BBB TJ proteins (n=3/group), L Nissl-stained hippocampal neurons, M Quantitative neuronal survival (n=3/group), N Brain inflammatory cytokines by ELISA (n=6/group), O Analysis of FITC-IPA fluorescence imaging in brain tissue, P CCK-8 cytotoxicity assay in HT22 cells (n=6/group), Q Inflammatory cytokines in HT22 cells by ELISA (n=6/group). $^{\#}P < 0.05$, $^{\#\#}P < 0.01$ versus CON group; $^{*}P < 0.05$, $^{**}P < 0.01$ versus DSS group.
